# Facile and Scalable Synthesis of Metal- and Nitrogen-Doped Carbon Nanotubes for Efficient Electrochemical CO_2_ Reduction

**DOI:** 10.1021/acssuschemeng.3c01222

**Published:** 2023-04-21

**Authors:** Yang Gang, John Pellessier, Zichen Du, Siyuan Fang, Lingzhe Fang, Fuping Pan, Manuel Suarez, Kirk Hambleton, Fan Chen, Hong-Cai Zhou, Tao Li, Yun Hang Hu, Ying Li

**Affiliations:** † J. Mike Walker ‘66 Department of Mechanical Engineering, Texas A&M University, College Station, Texas 77843, United States; ‡ Department of Materials Science and Engineering, Michigan Technological University, Houghton, Michigan 49931, United States; § Department of Chemistry and Biochemistry, Northern Illinois University, DeKalb, Illinois 60115, United States; ∥ Department of Chemistry, Texas A&M University, College Station, Texas 77843, United States; ⊥ Chemistry and Material Science Group, X-ray Science Division, Argonne National Laboratory, Lemont, Illinois 60439, United States

**Keywords:** commercial carbon nanotubes, metal−nitrogen coordinated single-atom catalyst, metal impurities in carbon nanotubes, scalable and sustainable catalyst synthesis, selective CO_2_ reduction to CO, flow cell testing, industrial relevant current, high stability

## Abstract

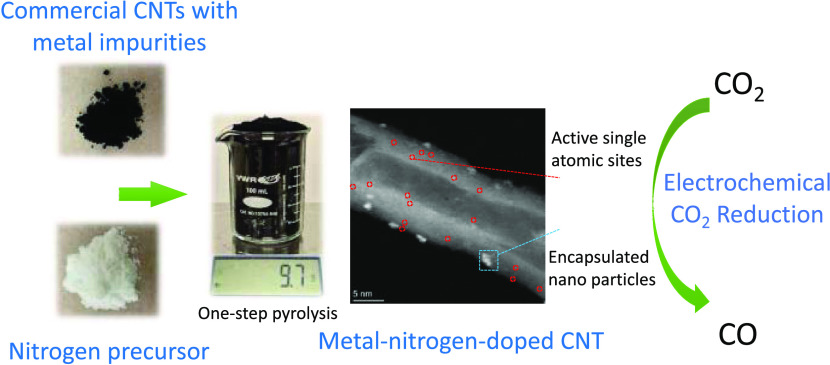

Metal- and nitrogen-doped carbon (M–N–C) is a promising material to catalyze electrochemical CO_2_ reduction reaction (CO_2_RR). However, most M–N–C catalysts in the literature require complicated synthesis procedures and produce small quantities per batch, limiting the commercialization potential. In this work, we developed a simple and scalable synthesis method to convert metal-impurity-containing commercial carbon nanotubes (CNTs) and nitrogen-containing organic precursors into M–N–C via one-step moderate-temperature (650 °C) pyrolysis without any other treatment nor the need to add metal precursors. Batches of catalysts in varied mass up to 10 g (150 mL in volume) per batch were synthesized, and repeatable catalytic performances were demonstrated. To the best of our knowledge, the 10 g batch is one of the largest batches of CO_2_RR catalysts synthesized in the literature while requiring minimal synthesis steps. The catalyst possessed single-atomic iron–nitrogen (Fe–N) sites, enabling a high performance of >95% CO product selectivity at a high current density of 400 mA/cm^2^ and high stability for 45 h at 100 mA/cm^2^ in a flow cell testing. The catalyst outperformed a benchmark noble-metal nanoparticle catalyst and achieved longer stability than many other reported M–N–C catalysts in the literature. The scalable and cost-effective synthesis developed in this work paves a pathway toward practical CO_2_RR applications. The direct utilization of metal impurities from raw CNTs for efficient catalyst synthesis with minimal treatment is a green and sustainable engineering approach.

## Abstract 2

Scalable synthesis of metal- and nitrogen-doped carbon nanotubes effective for electrochemical CO_2_ reduction is developed using two commercial raw materials under moderate-temperature pyrolysis.

## Introduction

Electrochemical CO_2_ reduction reaction (CO_2_RR) has appeared as a promising solution for mitigating atmospheric CO_2_ concentrations.^1,2^ Among different reaction pathways and products, CO_2_ to CO is one of the most practical solutions for future large-scale applications because a current density larger than 100 mA/cm^2^ and CO product selectivity larger than 90% have been widely demonstrated, and this pathway has lower production cost compared to other C1 or C2 products.^3−6^ Although extensive studies have been conducted to further improve CO selectivity and current density, the lack of mass production potential for synthesizing such catalysts and the associated high cost pose one of the biggest obstacles for industrial-scale CO_2_RR applications.^7,8^ Nobel metals such as Au and Ag were the first catalysts investigated by the research community, showing CO selectivity larger than 90%, but they are too expensive to scale up.^9,10^ Heteroatom-doped carbon-based catalysts, for example, iron- and nitrogen-doped graphene,^11^ nickel- and nitrogen-doped carbon black,^12^ and iron, nitrogen, and sulfur co-doped carbon nanosheets,^13^ have attracted increasing attention in recent years because of their lower costs and comparable catalytic performance to noble-metal catalysts.

Typically, the metal- and nitrogen-doped carbon (M–N–C) catalysts are synthesized by thermal treatment of a mixture of metal salts, carbon precursors, and nitrogen-containing compounds such as urea or melamine.^14^ During the pyrolysis, nitrogen species coordinate with metal atoms, forming a variety of functionalities (M–N*_X_*, *X* representing the coordination number) depending on the synthesis condition.^15−20^ These metal–nitrogen moieties are generally accepted as the active sites to facilitate CO_2_RR to CO.^21−23^ In addition, the physical properties of carbon supports, such as conductivity and surface area, as well as the interaction between M–N active sites and carbon supports are also essential to the M–N–C performance.^12,24−26^ To achieve high performance, a series of complex treatments are normally required in the synthesis process to obtain desired properties, which are potential barriers for large-scale synthesis.

Those treatments are typically categorized into four steps: carbon precursor preparation, mixing, pyrolysis, and post-pyrolysis treatment. First, two major routes are used to prepare the carbon precursor. One route begins with the synthesis of organic materials by polymerization or self-assembly (such as metal–organic frameworks ZIF-8, polyacrylonitrile, 3,4-ethylenedioxythiophene polymerization, etc.) to form the carbon precursor.^13,27,28^ Another route directly uses solid carbon allotropes such as carbon black, carbon nanotubes (CNTs), or graphite as the carbon precursor.^12,25,29,30^ These carbon precursors typically go through a series of activations and strong oxidation processes to grow oxygen-rich functional groups and enhance the surface area for the subsequent metal–nitrogen doping steps.^12,25,29−32^ The pretreated carbons, nitrogen precursors, and metal precursors are mixed by a variety of methods, including wet impregnation, freeze-drying, ball milling, etc.^13,33,34^ Then, the mixture undergoes high-temperature thermal treatment (usually around 800–1100 °C) to carbonize and form M–N active sites.^12,13,28,35,36^ As a final post-treatment step, acid washing is typically conducted to remove the excessive metal salts or metal nanoparticles. As demonstrated by the literature, metal nanoparticles facilitate the competing hydrogen evolution reaction (HER), thus decreasing CO_2_RR performance.^37^ Additional pyrolysis is usually conducted to remove the oxygen-containing groups generated by the acid washing to enhance the conductivity.^25,38−40^

The aforementioned multistep treatment and synthesis processes significantly hinder the potential for mass production of the M–N–C catalysts, despite the composing elements being more abundant and cheaper than precious metal Au or Ag catalysts. Efforts have been made to explore cheaper sources of precursors to reduce the overall cost. For example, M–N–C derived from biomass and industrial byproducts have been demonstrated to be active for CO_2_RR.^41−44^ However, these low-cost materials typically suffer from small geometric/electrochemical surface area and require extensive activation steps including high-temperature activation, concentrated acid washing, mixing with other high-surface-area carbon materials, etc. Therefore, there is an urgent demand to develop a method that utilizes commercially available materials, involves minimal treatment steps, and requires a milder synthesis condition than current state-of-the-art methods, while still ensuring high CO_2_RR activity from the synthesized catalysts.

In this work, we report a simple, facile, and scalable synthesis of M–N–C catalysts for efficient CO_2_ electrolysis by directly utilizing commercially available raw materials, multiwalled carbon nanotubes (MWCNTs) and melamine, which can be purchased in large quantities. Commercial MWCNTs were pyrolyzed under 650 °C with a nitrogen precursor (e.g., melamine) to synthesize single atomic M–N–C catalysts in one step, requiring no pre- or post-treatment steps. The metal impurities (e.g., Fe and/or Ni) already existing in the commercial CNTs serve as the metal sources and thus, no additional metal precursors are needed. Furthermore, the synthesis process can be extended to different types of commercial CNTs and a variety of nitrogen precursors, making it a versatile method. The as-prepared catalysts are evaluated in both H-Cell and flow cell electrolyzers under pure or low CO_2_ partial pressure environments. More than 90% CO_2_-to-CO selectivity at a commercially relevant current density (>100 mA/cm^2^) was achieved. Finally, the mass production capability is validated by simply scaling up the quantity of the precursor materials without any modifications to the synthesis parameters, resulting in different scales of catalyst mass from 0.1 to 10 g while maintaining high CO_2_RR performance.

## Experimental Section

### Catalyst Synthesis

#### CNT-Mel

Typically, 100 mg of raw multiwalled CNTs (>95 wt % purity, denoted as Raw-CNT) was mixed with 1.0 g of melamine by mortar and pestle. The powder mixture was then placed in a combustion boat and loaded into a tube furnace (Thermal Scientific, Lindberg Blue M). The sample was pyrolyzed in an Ar atmosphere at a flow rate of 80 standard cubic centimeters per minute (sccm) with a ramping rate of 5 °C/min until 650 °C and then maintained at this temperature for 3 h.

The as-prepared catalyst was denoted as CNT-Mel or CNT-Mel (650 °C). Two other samples were synthesized under the same condition except at different pyrolysis temperatures of 800 and 950 °C, they were denoted as CNT-Mel (800 °C) and CNT-Mel (950 °C), respectively.

To demonstrate a larger-scale synthesis capability, 500 mg of raw CNTs and 2.0 g of melamine were pyrolyzed at 650 °C for 3 h. The product was denoted as CNT-Mel-500 mg. Compared with CNT-Mel, the relative amount of melamine used for CNT-Mel-500 mg was reduced to lower the materials cost per unit mass of the catalyst. CNT-Mel-10 g was prepared using a similar method as CNT-Mel-500 mg, except using 10 g of commercial CNT and 40 g of melamine.

A control experiment using high-purity CNTs (>99.9% carbon) with a minimal amount of metal impurities, denoted as Pure-CNT, was also conducted to compare with Raw-CNT (>95% carbon). Pure-CNT was then doped with N through pyrolysis with melamine following the same procedure, and the sample is denoted as Pure-CNT-Mel.

#### CNT-Heat

A control sample was synthesized using the same pyrolysis process as CNT-Mel except that no melamine was added, and therefore no nitrogen doping was expected. The sample was donated CNT-Heat.

#### CNT-Mel-Acid

A control sample was synthesized by acid washing the CNT-Mel sample after pyrolysis, denoted as CNT-Mel-acid.

#### CNT-Urea and CNT-DY

CNT-Urea and CNT-DY were synthesized under the same condition as CNT-Mel except using urea or dicyandiamide as the nitrogen precursor, respectively, instead of using melamine.

#### CNT-Mel-V1 and CNT-Mel-V2

Raw CNTs from two different vendors were used to synthesize CNT-Mel-V1 and CNT-Mel-V2. Unless otherwise mentioned, the CNT-Mel presents CNT-Mel-V1 in this work, i.e., using CNTs from vendor V1. Same for the other catalysts including CNT-Urea and CNT-DY.

### Evaluation of CO_2_RR

#### H-Cell

The traditional H-Cell contains two compartments, separated by a proton exchange membrane (Nafion 115 membrane, Beantown Chemical, 0.125 mm thick). It is a system consisting of three electrodes, a working electrode (WE) and a reference electrode (RE: Ag/AgCl, 3 M KCl) at the cathode side, and a counter electrode (CE: 1 cm × 1 cm Pt foil) as the anode. The CO_2_-saturated 0.5 M KHCO_3_ solution was used as both catholyte and anolyte. The measured potentials after iR compensation are rescaled to the reversible hydrogen electrode by *E* (RHE) = *E* (Ag/AgCl) + 0.210 V + 0.0591 V × pH. The catalyst ink (3 mg of catalysts in a mixture of 370 μL of ethanol, 200 μL of water, and 30 μL of 5% Nafion solution) was sonicated for 3 h. The working electrode was prepared by drop-casting 200 μL of the catalyst ink onto a Toray carbon paper with an active catalytic geometric area of 1 cm^2^. High-purity CO_2_ (99.999%, Airgas) at a flow rate of 30 sccm was introduced in the cathode chamber for 30 min to fully saturate the catholyte and the flow rate was maintained throughout the test. The products were analyzed via an online gas chromatograph (GC, Fuel Cell GC-2014ATF, Shimadzu) equipped with a thermal conductivity detector (TCD) and a methanizer-assisted flame ionization detector (FID).

#### Flow Cell

Similar to our previous work, a customized flow cell electrolyzer was used to evaluate the feasibility of applying the catalyst at commercially viable current densities.^45^ The flow cell is a two-compartment system, consisting of anode and cathode chambers separated by an anion exchange membrane (Fumasep PK 130, Fuel Cell Stores). Nickel foam (active area: 1 cm^2^) was used as the anode for oxygen evolution reaction (OER), and the anolyte (1 M KOH) was circulated in the anode chamber at a flow rate of 10 sccm. The cathode was prepared by airbrushing the catalyst ink (10 mg catalyst, 3 mL ethanol, 300 μL of 5% Nafion solution) onto a gas diffusion layer (GDL) (Sigracet 39 BC, Fuel Cell Store) with a geometric area of 2 × 3 cm^2^ and was cut and used for the following tests. The catalyst was loaded to approximately 1 mg/cm^2^ based on the difference in electrode weight before and after airbrushing with an active area of 1 cm^2^. The catholyte (1 M KOH) was circulated in the cathode chamber between the membrane and cathode at a flow rate of 1.5 sccm. The CO_2_ gas, circulated at the backside of the GDL, diffused into the GDL and reacted at the catalyst–electrolyte interface. A Hg/HgO electrode (1 M KOH) was used as the reference. The flow cell tests were powered by a DC power supply (Agilent E3633A) and the potential between the reference and cathode was measured by a multimeter (AidoTek VC97+). All of the measured potentials were reported without iR compensation. The products in the flow cell systems were analyzed via an online gas chromatograph (GC, GC-2010, Shimadzu) equipped with a thermal conductivity detector (TCD) and a flame ionization detector (FID). Both CO and H_2_ were detected by the TCD, and methane and hydrocarbons were measured by the FID detector.

A varied CO_2_ partial pressure environment (75, 50, or 25%) was produced by diluting pure CO_2_ with N_2_ gas (Airgas, UHP grade). Unless otherwise indicated, the experiments were conducted in pure or 100% CO_2_ environment.

## Results and Discussion

### Morphology, Structure, and Composition

**Figure 1:**
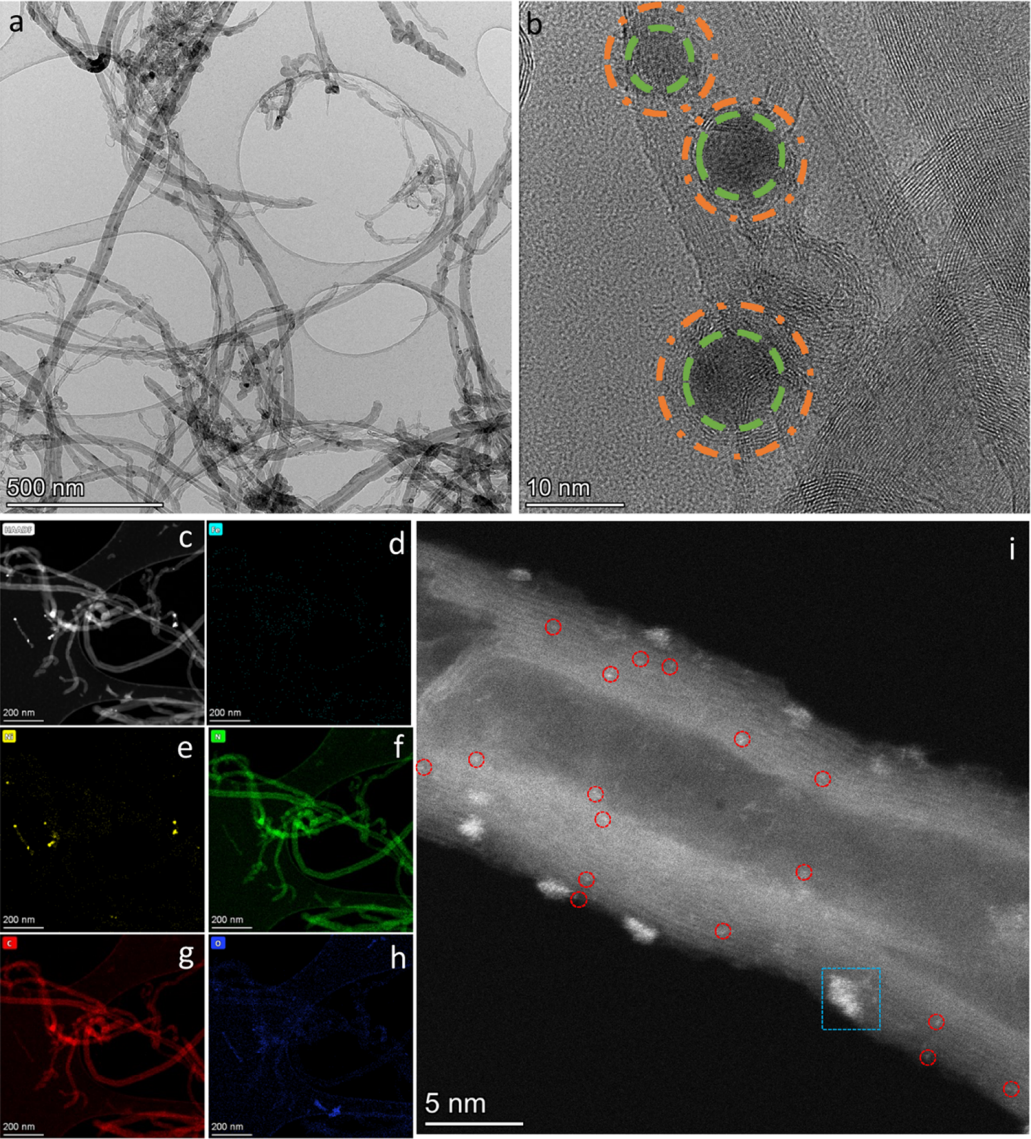
(a) TEM, (b) HR-TEM, (c) HAADF-STEM images and elemental mapping images of (d) Fe, (e) Ni, (f) N, (g) C, and (h) O of CNT-Mel, and (i) high-resolution HAADF-STEM images of the CNT branch of CNT-Mel.

Transmission electron microscopy (TEM) analyses were conducted to reveal the structure of Raw-CNT (Figure S1a,b), CNT-Mel (Figures 1a, and S1c,d), and CNT-Heat (Figure S1e,f). CNT-Mel and CNT-Heat maintain the tubular feature similar to Raw-CNT, with the presence of metal impurities in the form of either nanoparticle or nanocluster at varied sizes, indicating no major CNT morphology changes after pyrolysis with or without melamine. For CNT-Mel, no melamine residues are observed, indicating complete melamine decomposition. The metal impurities in the form of nanoparticles and nanoclusters are likely residues of metal catalysts used for manufacturing CNTs in the industrial process.^46^ The high-resolution TEM (HR-TEM) image in Figure 1b shows that metal nanoparticles are encapsulated by graphitic carbon layers. Exposed nanoparticles were not identified from HR-TEM, likely because the industrial purification process has removed the exposed nanoparticles by acid treatments, leaving the remaining nanoparticles encapsulated.^47−49^ The industrial purification process typically involves multiple physical/chemical steps including sonication, oxidation, acid washing, and thermal annealing.^48^ These treatments are proven to be effective for removing exposed metal impurities and amorphous carbons in the CNTs, thus generating high-purity products.^50^ Besides intrinsically encapsulated metal particles from the raw CNTs, there are still chances that the decomposition of melamine during the pyrolysis process in this work may result in additional encapsulation of metal impurities by N-doped carbon layers. It is challenging, though, to distinguish such sites from the original encapsulated metals in the pristine CNTs.

The dispersion of metal atoms was revealed by high-angle annular dark-field aberration-corrected scanning transmission electron microscopy (HAADF-STEM). First, the elemental mapping images demonstrate the uniform distribution of N and Fe dopants throughout the CNT branches (Figure 1c–h), but the distribution of Ni (Figure 1e) is much less uniform with clear and dense Ni aggregation, suggesting the formation of Ni nanoparticles on CNT-Mel. Furthermore, a higher-resolution STEM image of the CNT branch is shown in Figure 1i. Bright dots (circled in red, average diameter about 0.2 nm) are dispersed throughout the CNT branch, corresponding to single metal atoms.^51−53^ Besides the distribution of metal single atoms, nanoparticles (larger than 2 nm) and nanoclusters (less than 2 nm) are also observed on the CNT branch (Figure 1i). These results indicate that the metal impurities are in the form of both single atoms and nanoparticles/nanoclusters. In addition, the existence of bimetallic Fe–Ni sites are possible within nanoparticles/nanoclusters, given that Fe and Ni are the most popular metals used as the seeds for industrial and economical CNT manufacturing;^54,55^ however, the number of such sites should be small based on the elemental mapping results.

HAADF-STEM and elemental mapping were further conducted on the raw CNT (denoted as Raw-CNT) in Figure S2. Raw-CNT reveals similar metal structures to that of CNT-Mel, where aggregated Ni nanoparticles are clearly observed, and Fe elements are very well dispersed. A High-resolution STEM image of Raw-CNT (Figure S2g) also shows the existence of metal single atoms. These results confirm that commercial CNTs have intrinsic single atomic sites of metals, possibly in form of metal atoms coordinate with C and/or O, as reported in the literature.^56^ These single atomic sites are likely to coordinate with nitrogen during pyrolysis with organic precursors to form M–N–C sites.^57^

**Figure 2:**
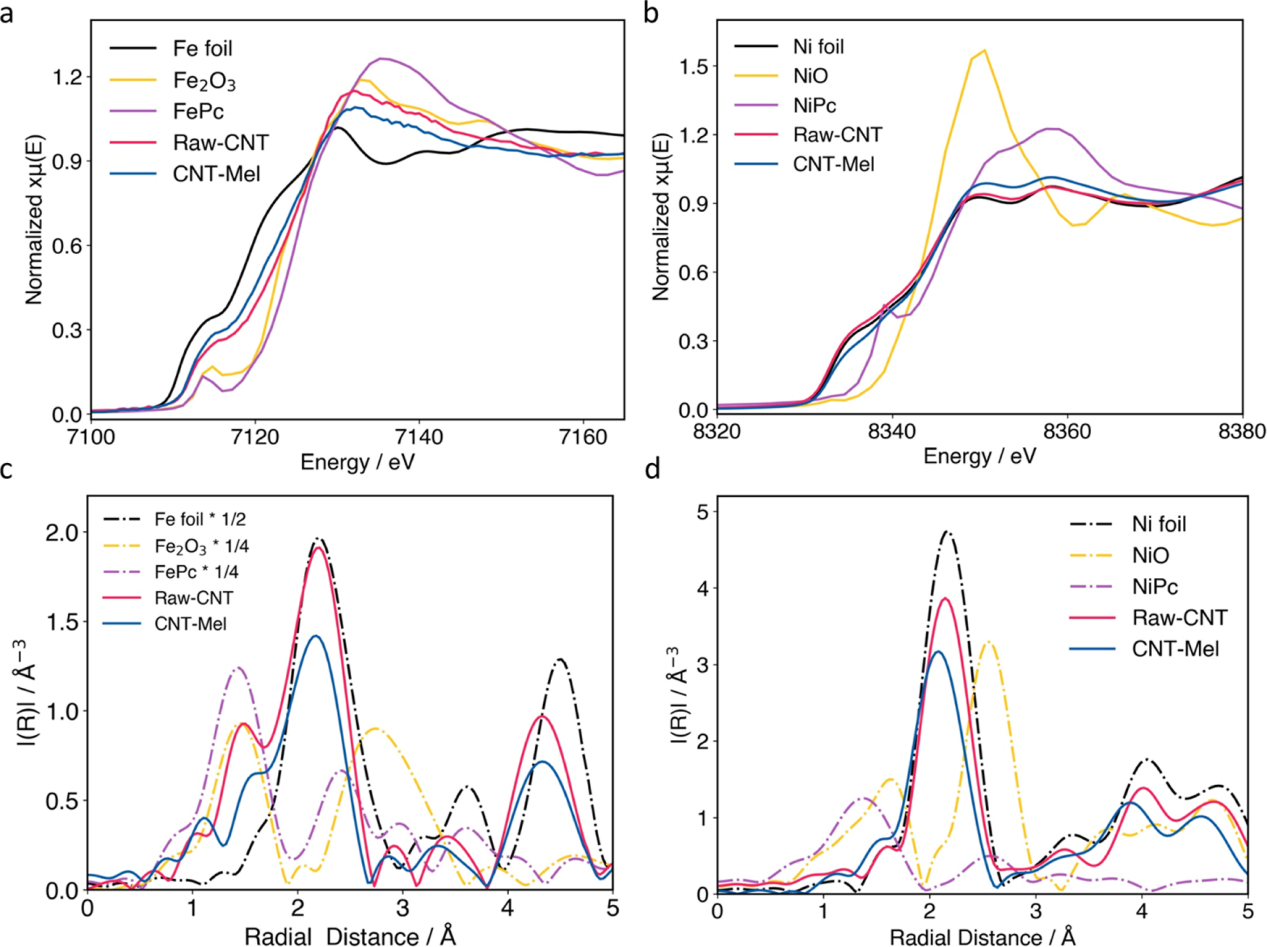
(a) Fe X-ray absorption near-edge structure (XANES), (b) Ni XANES, and Fourier transform of the (c) Fe extended X-ray absorption fine structure (EXAFS) spectra and (d) Ni EXAFS spectra of Raw-CNT, CNT-Mel, and standard references.

X-ray diffraction (XRD) was conducted to analyze the crystal structure of the catalysts. As shown in Figure S3, all samples reveal similar diffraction patterns, indicating a similar crystal structure. A major peak at 26° corresponds to C(002). A broad peak consisting of two minor peaks between 42 and 43° corresponds to the two-dimensional (2D) carbon lattice, C(100) and C(101), respectively, agreeing with a typical carbon nanotube XRD pattern.^58,59^ A minor peak close to 44° could be assigned to either Ni(111) or Fe(110) since they overlap.^60,61^ However, from scanning transmission electron microscopy/energy dispersive X-ray spectroscopy (STEM/EDS) (Figure 1), Ni elements exist primarily as nanoparticles, while Fe elements dominate the single atom sites; thus, the peak at 44° more likely corresponds to Ni metal nanoparticles because Fe single atomic sites cannot be detected by XRD.^62^ The existence of bimetallic FeNi cannot be ruled out because the FeNi peak overlaps with C(100) peak that is close to 42°; however, the quantity of such bimetallic sites is small, if any, because no major NiFe peaks are observed, unlike those reported in the literature.^63−65^ This is consistent with the observations by STEM/EDS (Figure 1) and X-ray absorption spectroscopy (XAS) (Figure 2) analyses, which demonstrate most of the Ni elements exist in the system as nanoparticles encapsulated by carbon layers.

To further understand the surface element composition, X-ray photoelectron spectroscopy (XPS) was conducted. As revealed in Table S1, the surface N content decreases with the pyrolysis temperature, from 1.3 atom % on CNT-Mel (650 °C) to 0.7 atom % on CNT-Mel (800 °C) and 0.6 atom % on CNT-Mel (950 °C). This is possible because the nitrogen doping sites are less stable at a higher pyrolysis temperature.^66^ The surface Fe concentrations of the three samples are in the similar range of 0.4–0.5 atom %, regardless of the pyrolysis temperature. The surface Ni concentration is much less than that of Fe, with less than 0.1 atom % at 650 °C and almost undetectable at 800 and 950 °C. Because XPS is only sensitive to a depth of 5–10 nm from the surface,^45,67,68^ the result that Ni is at an extremely low content or even not detected by XPS further confirms that the majority of Ni elements are encapsulated by carbon layers and more Fe elements are exposed on the surface as single atom sites, which is consistent with the findings from the TEM/STEM analyses. These findings are also consistent with the theoretical calculation from the literature that Fe elements are stable as isolated atoms on the graphitic carbon surface while Ni tends to diffuse instantaneously at a high temperature.^69^ Furthermore, N 1s spectra are fitted to analyze the surface N composition, as shown in Figure S4. Four possible types of nitrogen species are fitted to the raw data, pyridinic N (398.2 eV), pyrrolic N (399.5 eV), graphitic N (401.3 eV), and N oxides (403 eV).^23,70^ The first three N species are found in the system, with pyrrolic N having the largest content. As pyrolysis temperature increases, the overall nitrogen peak becomes smaller with larger noises, representing the decrease of surface nitrogen content, consistent with the literature.^71^ Regardless of the pyrolysis temperature, the edge-located N species (pyrrolic N and pyridinic N) are dominating in CNT-Mel that contribute to active M–N sites responsible for catalyzing CO_2_ to CO reduction.^72,73^

X-ray absorption spectroscopy (XAS) was performed on Raw-CNT and CNT-Mel to compare their local arrangements of Ni and Fe atoms. Fe foil, Fe_2_O_3_, iron phthalocyanine (FePc), Ni foil, NiO, and nickel phthalocyanine (NiPc) were used as the standard references. As shown in Figure 2a, X-ray absorption near-edge structure (XANES) spectra, the Fe adsorption edge profiles of Raw-CNT and CNT-Mel are between Fe foil and Fe_2_O_3_, suggesting the oxidation state of these samples between 0 and +3. This is in agreement with the literature that single atomic sites of Fe typically possess an oxidation state between metallic (0) and fully oxidized state (+3).^46,74,75^ In CNT-Mel, this could be also due to the formation of Fe–N bonds, resulting in the increase of the oxidation state of the transition metals.^12,76^ In contrast, the XANES spectra of Ni edges of Raw-CNT and CNT-Mel in Figure 2b reveal almost identical edge profiles, all close to that of Ni foil. This again confirms the oxidation states of Ni in both samples are close to 0.

**Figure 3:**
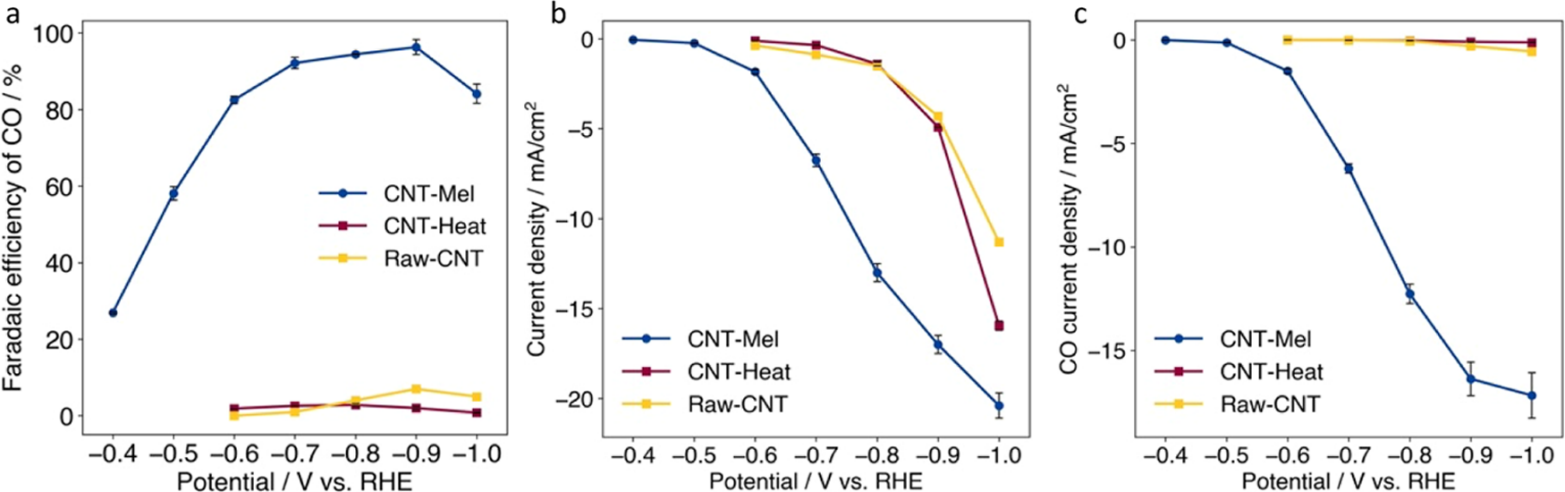
(a) Faradaic efficiency of CO, (b) current density, and (c) CO partial current density of CNT-Mel, nitrogen-free control sample CNT-Heat, and Raw-CNT in H-Cell testing (electrolyte: 0.5 M KHCO_3_).

Furthermore, extended X-ray absorption fine structure (EXAFS) was analyzed to uncover the coordination environment of Fe (Figure 2c) and Ni (Figure 2d) atoms. In Figure 2c, both Raw-CNT and CNT-Mel show a peak at around 1.4 Å, which could be assigned to Fe–C, Fe–O (as in Fe_2_O_3_), and/or Fe–N (as in FePc^25^) since they all have a similar bond length.^77,78^ Since the N element is not present on Raw-CNT according to XPS (Table S1) and EDS (Figure S2) results, it is likely that Fe–C or Fe–O sites (due to oxidation of CNTs) exist on Raw-CNT. On the other hand, it is more likely that CNT-Mel is rich in Fe–N sites since N-doping in CNT-Mel is clearly evidenced by EDS (Figure 1f) and XPS (Table S1) results; possible minor Fe–C or Fe–O sites may exist on CNT-Mel as well. However, based on the CO_2_RR performance of Raw-CNT as shown in Figure 3, almost no CO production is observed, indicating the presence of Fe–C or Fe–O, if any, makes little contribution to CO_2_ conversion to CO, agreeing with the literature finding of the Fe–C or Fe–O activity.^79,80^ Given all the evidence that Fe is atomically dispersed (STEM/EDS), Fe content on the surface is much higher than Ni (XPS), Fe oxidation state is in between 0 and 3 (EXANES), and Fe–N bonds are identified (EXAFS), it is concluded that Fe elements are present primarily as single atoms coordinated with N to form Fe–N sites on CNT-Mel. It is noticed that a peak at around 2.1 Å also exists in both Raw-CNT and CNT-Mel, likely corresponding to Fe–Fe or Fe–Ni bond.^81−83^ These bonds possibly exist inside the encapsulated nanoparticles or nanoclusters from the pristine CNTs.

The EXAFS plots of Ni edges in Raw-CNT and CNT-Mel in Figure 2d show a predominate peak at 2.1 Å, corresponding to the Ni–Ni peak. Both samples reveal almost identical Ni bonding environments to that of Ni foil, while no obvious Ni–N bonds are observed. Combing the result that the Ni oxidation states are all close to 0 (Figure 2b), it is confirmed that the Ni elements in these materials are primarily in the form of nanoclusters/nanoparticles encapsulated in carbon layers as shown in Figure 1. Although the existence of single atomic Ni–N sites cannot be fully excluded, the lack of Ni–N bond from XAS and much less surface distribution of Ni than Fe from XPS suggest the quantity of such is small.

In summary, the above characterization results suggest the existence of both Fe and Ni elements in CNT-Mel. Both single atomic sites and metal nanoparticles/nanoclusters are observed in the system. Single atomic sites are mostly Fe–N sites while Ni exists primarily as metal nanoparticles/nanoclusters encapsulated by the graphitic carbon layers.

### CO_2_RR Performance Evaluation in H-Cell

The electrochemical CO_2_ reduction performance of CNT-Mel and CNT-Heat (no nitrogen doping) were evaluated first in a three-electrode H-Cell reactor. Synthesis of CNT-Mel at different pyrolysis temperatures (650, 800, and 900 °C) was conducted to investigate the optimal synthesis condition (Figure S5). From Figure S5a,b, the three samples do not exhibit very different FE(CO) and total current density, but CNT-Mel (650 °C), i.e., CNT-Mel in previous sections, shows a larger CO current density than the samples prepared at higher temperatures over the entire potential range tested (Figure S5c). The lower CO_2_RR performance for higher pyrolysis temperature samples may be due to the decreased surface nitrogen content, as revealed by the XPS results (Table S1), which may have led to a reduced number of M–N sites. We attempted to further decrease the pyrolysis temperature to 550 °C, but a certain amount of the melamine precursor did not fully decompose, leading to a dark brown colored product instead of black CNT-based catalysts. As a result, 650 °C was identified as the optimal pyrolysis temperature, which is lower than those reported in many other studies required for producing single atomic M–N–C catalysts using other precursors and methods (Table S2). This is another advantage of the synthesis method reported in this work.

The Faradaic efficiency of CO (FE(CO)), total current density, and the partial CO current density of the optimized sample CNT-Mel (corresponding to 650 °C pyrolysis temperature if not mentioned otherwise) are shown in Figure 3. As revealed in Figure 3a, FE(CO) of CNT-Mel reached a high value (above 90%) in a wide potential range, from −0.7 to −0.9 V vs RHE, and the current density of CO reached 12.3 mA/cm^2^ at −0.8 V vs RHE (Figure 3b,c). In contrast, the control sample CNT-Heat and Raw-CNT had near zero FE(CO) and CO current density, and the total current produced was exclusively attributed to hydrogen evolution (Figure 3). In the CO_2_RR process, it has been widely accepted that the single atomic metal–nitrogen active sites (primarily Fe–N and Ni–N) are efficient to facilitate the formation of COOH* intermediates and the desorption of CO*.^12,46,84−86^ The much lower reaction rate of the control CNT-Heat and Raw-CNT samples than that of CNT-Mel is aligned with the literature findings. This also indirectly proves that the single atomic sites that exist in CNT-Mel are mostly Fe–N instead of Fe–O or Fe–C that exists in Raw-CNT. In addition, no CO_2_RR performance is observed with plain carbon paper, as shown in Figure S6. Furthermore, the carbon in the CO product has been widely proven to originate from CO_2_ instead of carbon catalyst or carbon paper through isotope studies in the literature.^87−90^ Thus, the CO produced in this work is from CO_2_ not from carbon content in the catalyst or carbon paper.

To better understand the active sites, poisoning experiments were conducted using ethylenediaminetetraacetic acid (EDTA) and potassium thiocyanate (KSCN).^91,92^ As shown in Figure S7, CNT-Mel with both KSCN and EDTA poisoning shows a reduced CO_2_RR performance, indicating the poisoning effect on the active sites. According to the literature, EDTA tends to bind only with single atomic sites, and thus, the reduction of CO_2_RR performance of both Faradaic efficiency of CO and CO partial current density in CNT-Mel-EDTA suggests single atomic site being the major contributor to the high CO_2_RR performance.^91,92^ Moreover, the literature indicates that KSCN tends to bind with both metal single atomic sites and metal nanoparticles, and thus a higher poisoning effect by KSCN than by EDTA would be anticipated if metal nanoparticles contribute to the electrochemical performance.^92^ The similar performances of CNT-Mel-EDTA and CNT-Mel-KSCN in Figure S7 indicate minimal contribution from metal nanoparticles in CNT-Mel, likely because these metal nanoparticles are encapsulated by carbon layers and are inaccessible to the poisoning reagents. This conclusion is further confirmed by comparing the activity of CNT-Mel with the acid-washed sample, the latter of which should have no exposed metal nanoparticles. As shown in Figure S8, CNT-Mel and CNT-Mel-acid samples show similar CO_2_RR performance, indicating no significant amount of exposed metal nanoparticles on the catalyst surface.

To study whether N-doped CNTs (without single metal atoms) are active for CO_2_RR, we have conducted a control experiment using high-purity CNTs (>99.9% carbon) from the same vendor that has minimal metal impurity (Pure-CNT) to compare with the Raw-CNT (>95% carbon) that has up to 5% metal impurity. Pure-CNT was then doped with N through pyrolysis with melamine to form Pure-CNT-Mel following the same procedure as preparing CNT-Mel. As shown in Figure S9, almost no activity of CO_2_RR was found in Pure-CNT-Mel, indicating N-doping alone without metal on CNTs has little contribution. Meanwhile, Raw-CNT and CNT-Heat samples, both having metal impurities but no N dopants, showed little CO_2_RR activity as well. These results confirm the importance of having both metal and nitrogen doping to activate CO_2_RR.

There is one scenario that metal nanoparticles could possibly contribute to CO_2_RR when the encapsulating carbon layer is composed of single atomic M–N sites or N-doped carbon. Such metal nanoparticles coupled with pyrrolic N species (Figure S4) have been found effective to resist the poisoning test.^93,94^ As also revealed in our prior work,^95^ through density functional theory (DFT) calculations, there exists a synergetic effect from Fe–N sites and encapsulated Ni nanoparticles that enhances CO_2_RR performance by reducing the desorption energy of *CO intermediates. Such a synergy may exist on the CNT-Mel catalyst in this work, but it is experimentally challenging to decouple this synergistic effect from the single atomic sites alone, which is believed to be the major contributor to the catalytic performance.

It should be noted that because of the coexistence of Fe and Ni impurities of commercial CNTs with atomic metal forms being mostly from Fe elements and Ni nanoparticles being encapsulated in carbon, it is impossible to completely remove one of the metals to form pure Fe–N–C or Ni–N–C catalysts as control samples to compare with the CNT-Mel where Fe and Ni coexist. Nevertheless, the characterization, various control experiments, and poisoning-experiment results in correlation with the activity data suggest the major contribution to CO_2_RR performance is from Fe–N single atomic sites on CNTs and possible secondary contribution is from the synergetic effect between Fe–N or N-doped carbon and the encapsulated Ni NPs, as reported in the literature and our prior theoretical work.^94,95^

### CO_2_RR Performance in Flow Cell in Different CO_2_ Partial Pressure Environments

#### CO_2_RR Performance at Different Current Densities

**Figure 4:**
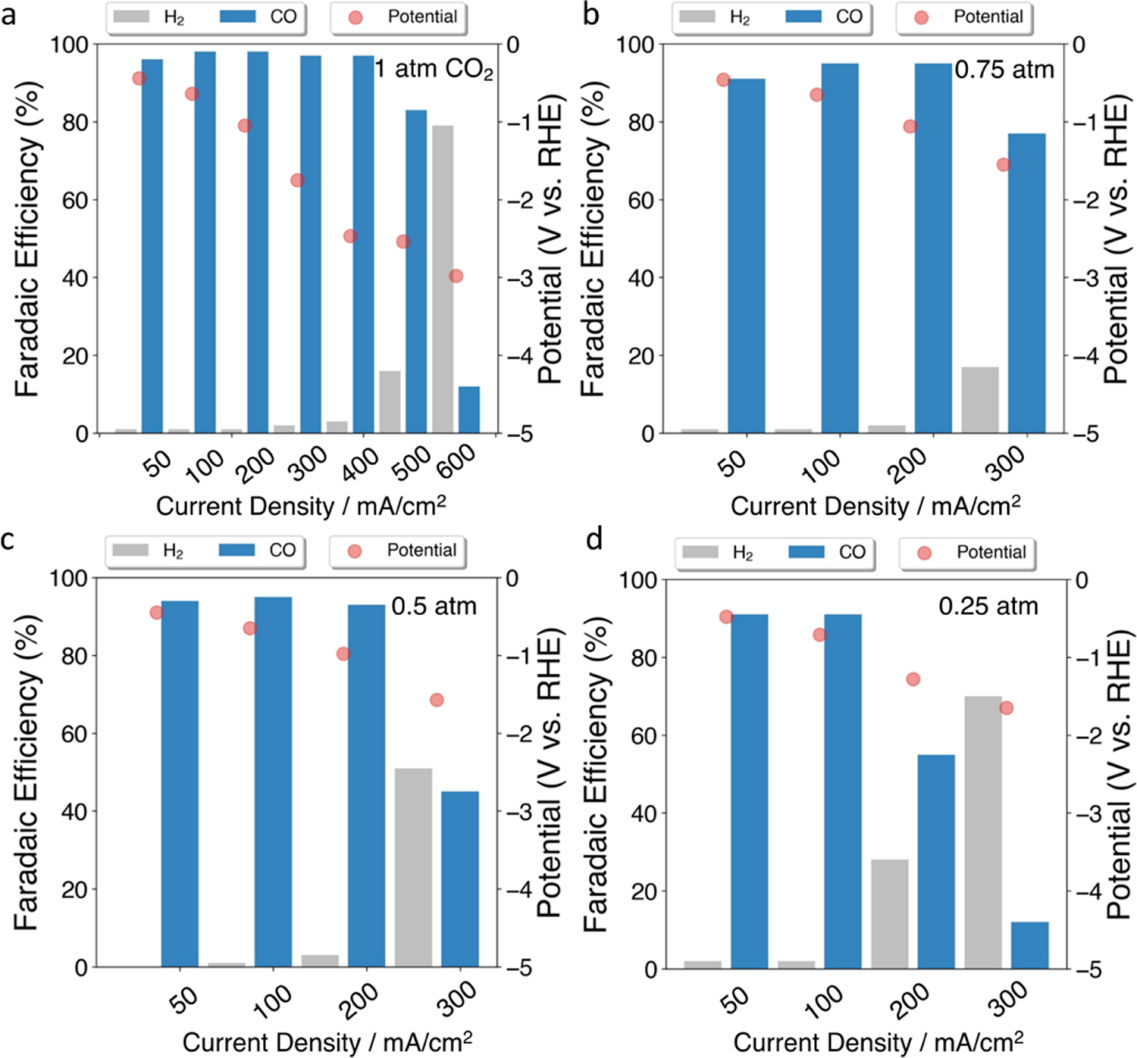
Product selectivity and cathode potential of CNT-Mel tested at different current densities under varied CO_2_ partial pressures ((a) 1 atm; (b) 0.75 atm; (c) 0.5 atm; (d) 0.25 atm) in the flow cell (electrolyte: 1 M KOH).

As revealed in Figure 4a, the selectivity of CO remained above 90% at a wide range of current densities, from 50 to 500 mA/cm^2^. Specifically, the Faradaic efficiency of CO is 98% at 100 mA/cm^2^ and 97% at 400 mA/cm^2^. The cathode potential is measured to be −0.64 V vs RHE without iR compensation at 100 mA/cm^2^ with a total cell voltage of 2.98 V. These results indicate commercially viable current densities.^4^

Furthermore, different concentrations of CO_2_ sources were used for the flow cell tests to study the effects of CO_2_ partial pressure. As revealed in Figure 4, four samples show similar cathode potentials, indicating that no additional energy input is required when switching to the diluted environment. However, the CO selectivity starts to decrease to around 77% at a current density of 300 mA/cm^2^ in 75% CO_2_ environment (Figure 4b). In Figure 4c, the CO selectivity in 50% CO_2_ environment reaches around 45% at 300 mA/cm^2^. In 25% CO_2_ environment (Figure 4d), the CO selectivity is around 12% at 300 mA/cm^2^. These results show a trend that the electrolysis starts to generate more H_2_ and less CO at a lower current density, as feed CO_2_ concentration decreases. This is likely because smaller areas of the catalyst surface are covered by CO_2_ in a more diluted environment, leading to a higher HER reaction rate at higher current densities.

#### CO_2_RR Stability Tests

**Figure 5:**
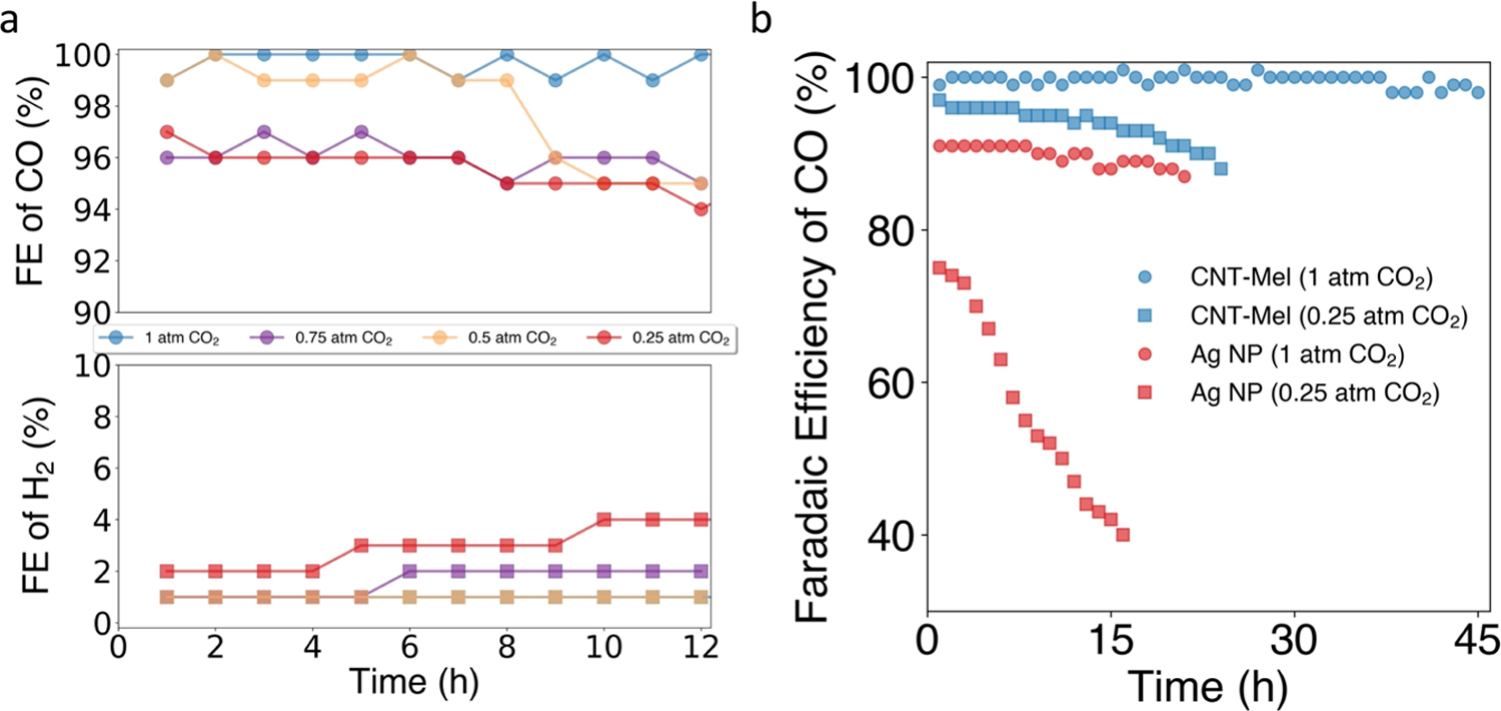
(a) Product selectivity of CNT-Mel stability tests at different CO_2_ partial pressures and (b) comparison of CNT-Mel with the benchmark catalyst Ag NP in flow cell testing (electrolyte: 1 M KOH; current density: 100 mA/cm^2^).

To demonstrate the stability of CNT-Mel, longer-term tests at different CO_2_ partial pressure environments were conducted at a current density of 100 mA/cm^2^. As revealed in Figure 5a, the performance of CNT-Mel does not show an obvious decrease in the 12 h test at all four CO_2_ concentrations, maintaining a high FE(CO) above 90%. In an even longer-term testing as shown in Figure 5b, CNT-Mel shows stable performance with more than 95% FE(CO) for 45 h in 100% (1.0 atm) CO_2_ environment, while in 0.25 atm CO_2_ environment, the CO selectivity slightly decreases from 99 to 90% after 24 h. For comparison, a benchmark catalyst, commercial silver nanoparticles (Ag NPs) were tested, which showed a lower stability in both 1.0 and 0.25 atm CO_2_ environments. In particular, the stability of Ag catalyst in 0.25 atm CO_2_ is much worse, with FE(CO) dropping from 76 to 39% in 24 h. This demonstrates the significant advantage of the prepared M–N–C catalysts as a cost-effective solution for CO_2_RR in large-scale applications. The stability of cathode potential is shown in Figure S10. The cathode potentials of all samples (including Ag catalyst) showed a slight increase at about 0.1 V in 12 h. In a longer-term 45 h test of CNT-Mel, the potential increased by about 0.2 V and appeared to level off after 24 h. It is worth noting that all samples showed the same trend, independent of the catalytic selectivity performance as shown in Figure 5, suggesting this is unlikely a catalyst-specific issue. The literature has pointed out common issues in alkaline CO_2_ flow cells caused by (bi)carbonates formation during the electrolysis. Generation of these (bi)carbonates consumes OH^–^ and increases the cathode impedance.^96−99^ Separate studies on electrolyzer design are needed to overcome the practical challenges in large-scale CO_2_RR application.

### Universality and Scalability in Materials Synthesis

#### Investigation of the Method Universality By Using Different Types of Nitrogen Precursors and Brands of CNTs

**Figure 6:**
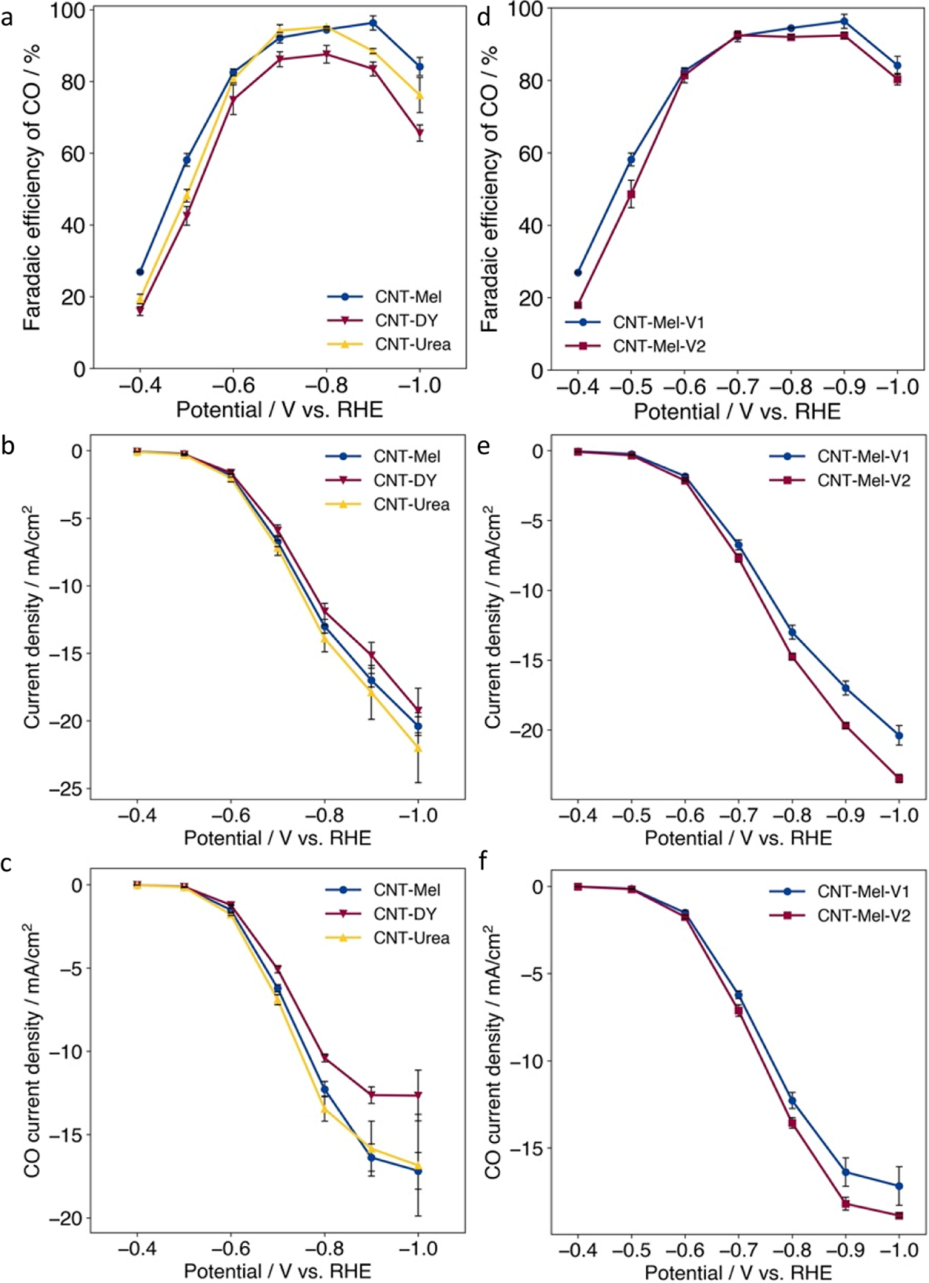
(a) Faradaic efficiency, (b) total current density, and (c) CO current density of catalysts synthesized from different nitrogen precursors; (d) Faradaic efficiency, (e) total current density, and (f) CO current density of catalysts synthesized from different CNT vendors, all in H-Cell testing (Electrolyte: 0.5 M KHCO_3_).

To further demonstrate the universally applicable nature of the synthesis method in this work, catalysts were prepared using two other widely used short-chain nitrogen precursors, urea and dicyandiamide. As shown in Figure 6a–c, CNT-Mel and CNT-Urea demonstrated comparable CO_2_RR performance and were slightly better than CNT-DY. All three samples had much higher performance than the control sample CNT-Heat that had no nitrogen doping (Figure 3). This result indicates that the developed method in this work can be extended to many other nitrogen precursors to generate the M–N–C catalysts based on commercial CNTs.

Furthermore, commercial CNTs from two vendors were used to synthesize CNT-Mel-V1 and CNT-Mel-V2 and their CO_2_RR performances were compared. As shown in Figure 6d–f, both catalysts show similar results in terms of FE(CO) and current density. This indicates the synthesis method developed in this work can be extended to commercial CNTs from different manufacturers, besides the flexibility of nitrogen precursors.

#### Investigation of Scalability of Synthesis and Repeatability of Performance

**Figure 7:**
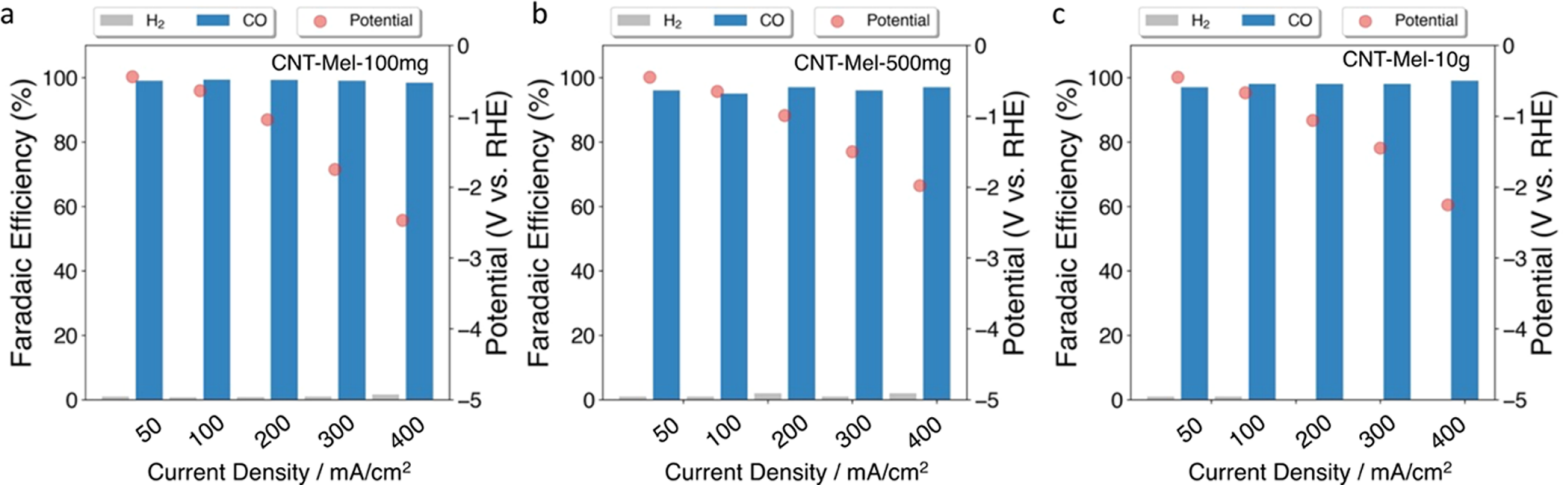
CO_2_RR performance of (a) CNT-Mel-100 mg, (b) CNT-Mel-500 mg, and (c) CNT-Mel-10 g in the flow cell (electrolyte: 1 M KOH).

To further demonstrate the scalability of the synthesis method in this work, a series of batches with different masses (0.1, 0.5, and 10 g) were synthesized, denoted by CNT-Mel-100 mg, CNT-Mel-500 mg, and CNT-Mel-10 g, respectively. CNT-Mel-100 mg, i.e., CNT-Mel denoted elsewhere in the work, set the baseline for the catalyst performance, as shown in Figure 3, and is later compared with the other two later batches. The melamine/CNT mass ratio was also reduced from 10 to 4 in the larger two batches to investigate the possibility of saving nitrogen precursors. Figure S11 shows the photo of the 10 g batch, which corresponds to approximately 150 mL in volume. As revealed in Figure S12 H-cell testing, the CO_2_RR performances of both CNT-Mel-500 mg and CNT-Mel-10 g are comparable to that of CNT-Mel-100 mg, especially at the optimum potential range (−0.6 to −0.8 V vs RHE), indicating the scalability of the synthesis method with good catalytic performance maintained. Furthermore, the flow cell testing results as shown in Figure 7 indicate that all three samples (CNT-Mel-100 mg, CNT-Mel-500 mg, and CNT-Mel-10 g) had similar CO_2_RR performance (>95% FE CO) in the current density range from 50 to 400 mA/cm^2^.^100^ This manufacturing method is further scalable by using larger apparatus such as a larger mixer and pyrolysis furnace because it only requires simple mixing and pyrolysis of commercial raw materials, suggesting a great potential for mass production to meet industrial needs.

#### Comparison to the Literature

We further compared the synthesis method and CO_2_RR performance to the literature. As shown in Table S2, regarding H-Cell performance, the catalyst prepared in this work is among the top-level performances for Fe-based and Ni-based catalysts, with this method requiring significantly fewer treatment steps under milder synthesis conditions. In addition, as shown in Table S3, the synthesis method achieves one of the largest batches reported in the literature, indicating a significant advantage of this work on potential future mass applications. As listed in Table S4, regarding the flow cell performance, the catalyst synthesized in this work demonstrates one of the best performances while the duration of our stability tests is longer than those in the literature.

## Conclusions

In conclusion, a facile, simple, and highly scalable method was developed in this work using two types of commercial materials, CNT and a N-containing organic precursor. Unlike conventional synthesis methods, this method required only one-step pyrolysis of the mixture at 650 °C without the need of any pre- or post-treatment. It is also applicable to different types of nitrogen precursors and different brands of CNTs, indicating significant universality for different raw materials. In addition, different batches in mass (0.1 to 10 g) were synthesized and the catalytic performances were comparable, indicating the significant scale-up potential. From STEM/XAS investigation, both Ni and Fe metal impurities exist in the raw CNTs and are preserved after pyrolysis while the metals are coordinated with N dopants to form M–N–C catalysts. The Ni metals primarily exist as nanoparticles/nanoclusters that are encapsulated by carbon layers. Single atomic Fe–N sites are believed to be the main active sites responsible for the high CO_2_RR performance observed in this work, with the possibility of minor contribution from the synergetic effect of Ni NP and Fe–N. The prepared catalysts have achieved more than 95% CO selectivity and demonstrated long-term stability of 45 h at 100 mA/cm^2^ in a pure CO_2_ environment, outperforming the benchmark Ag NP catalyst and other single atomic M–N–C catalysts, ranking among the top of the leading literature. The catalyst also shows much more stable performance at a diluted CO_2_ environment (25%) than that of Ag NP, achieving >90% CO selectivity for 24 h at 100 mA/cm^2^, indicating the feasibility in potential practical applications. The findings in this work provide a viable solution to cost-effective CO_2_RR at a large scale by developing a facile and scalable catalyst synthesis method.

## Supporting Information Available

Product selectivity calculation; equipment for materials characterization; additional results of TEM, HAADF-STEM, XRD, and XPS analyses; additional results on H-Cell performances (Faradaic efficiency of CO, total current density, and current density of CO) of the following samples: catalysts synthesized at different pyrolysis temperatures, plain carbon paper, samples after poisoning experiments, samples after acid washing, high-purity CNTs without metal impurities, and catalysts synthesized from different mass batches (0.1, 0.5 and 10 g); stability of cathode potential in flow cell testing; photo of synthesized 10 g batch catalysts; performance comparison between the catalyst in this work with those in the leading literature in both H-Cell and flow cell; and comparison of mass of catalysts synthesized in one batch with the literature (PDF)
