# Supplementary material for: Facile and Scalable Synthesis of Metal- and Nitrogen-Doped Carbon Nanotubes for Efficient Electrochemical CO2 Reduction
Source: ACS Sustain Chem Eng. 2023 Apr 21;11(18):7231–43. doi: 10.1021/acssuschemeng.3c01222 (PMC10853974; doi:10.1021/acssuschemeng.3c01222)
Supplement: Supplementary file 1 — sc3c01222_si_001.pdf [file sc3c01222_si_001.pdf]

Supporting Information

Facile and Scalable Synthesis of Metal and Nitrogen-Doped Carbon Nanotubes for Efficient Electrochemical CO_2 Reduction

Yang Gang[1], John Pellessier[1], Zichen Du[1], Siyuan Fang[2], Lingzhe Fang[3], Fuping Pan[1], Manuel Suarez[1], Kirk Hambleton[1], Fan Chen[4], Hong-Cai Zhou[4], Tao Li[3, 5], Yun Hang Hu[2], and Ying Li[1*]

[1] J. Mike Walker '66 Department of Mechanical Engineering, Texas A&M University, College Station, Texas, 77843, United States

[2] Department of Materials Science and Engineering, Michigan Technological University, Houghton, Michigan 49931, United States

[3] Department of Chemistry and Biochemistry, Northern Illinois University, DeKalb, Illinois 60115, United States

[4] Department of Chemistry, Texas A&M University, College Station, Texas, 77843, United States

[5] Chemistry and Material Science Group, X-ray Science Division, Argonne National Laboratory, Lemont, Illinois 60439, United States

* Corresponding author email: yingli@tamu.edu

Number of Pages: 24
Number of Figures: 12
Number of Tables: 4

Product Selectivity Calculation

The Faradaic efficiency (FE) of gaseous products in H-cell setup and flow cell setup at each applied potential was calculated based on the equation:

$$FE = \frac{z \cdot P \cdot F \cdot V \cdot v_i}{R \cdot T \cdot J}$$

Where z is the number of electrons transferred per mole of gas product (z is 2 for CO and H_2), P is pressure (1.01×10^5 Pa), F is Faraday constant (96500 C mol^{-1}), V is the gas volumetric flow rate (5.67×10^{-7} m^3/s), v_i is the volume concentration of gas product determined by GC, R is the gas constant (8.314 J/mol·K), T is the temperature (298.15 K), and J is the steady-state current at each applied potential (A).

The flow rate V was measured by a bubble flow meter (Gilian Gilibrator 2) at the gas outlet in the cathode chamber to consider the potential flow rate loss from CO_2 dissolving in the alkaline electrolyte.

Equipment for Materials Characterizations

Morphology, structure, and composition of the catalysts were characterized by transmission electron microscopy (TEM, FEI Tecnai G2 F20 ST), high-angle angular dark-field scanning transmission electron microscopy (Hitachi 2700C), and X-ray photoelectron spectroscopy (XPS, Omicron). The X-ray absorption spectroscopy (XAS) measurements were performed at the 12-BM beamline of the Advanced Photon Source (APS) at the Argonne National Laboratory (ANL).

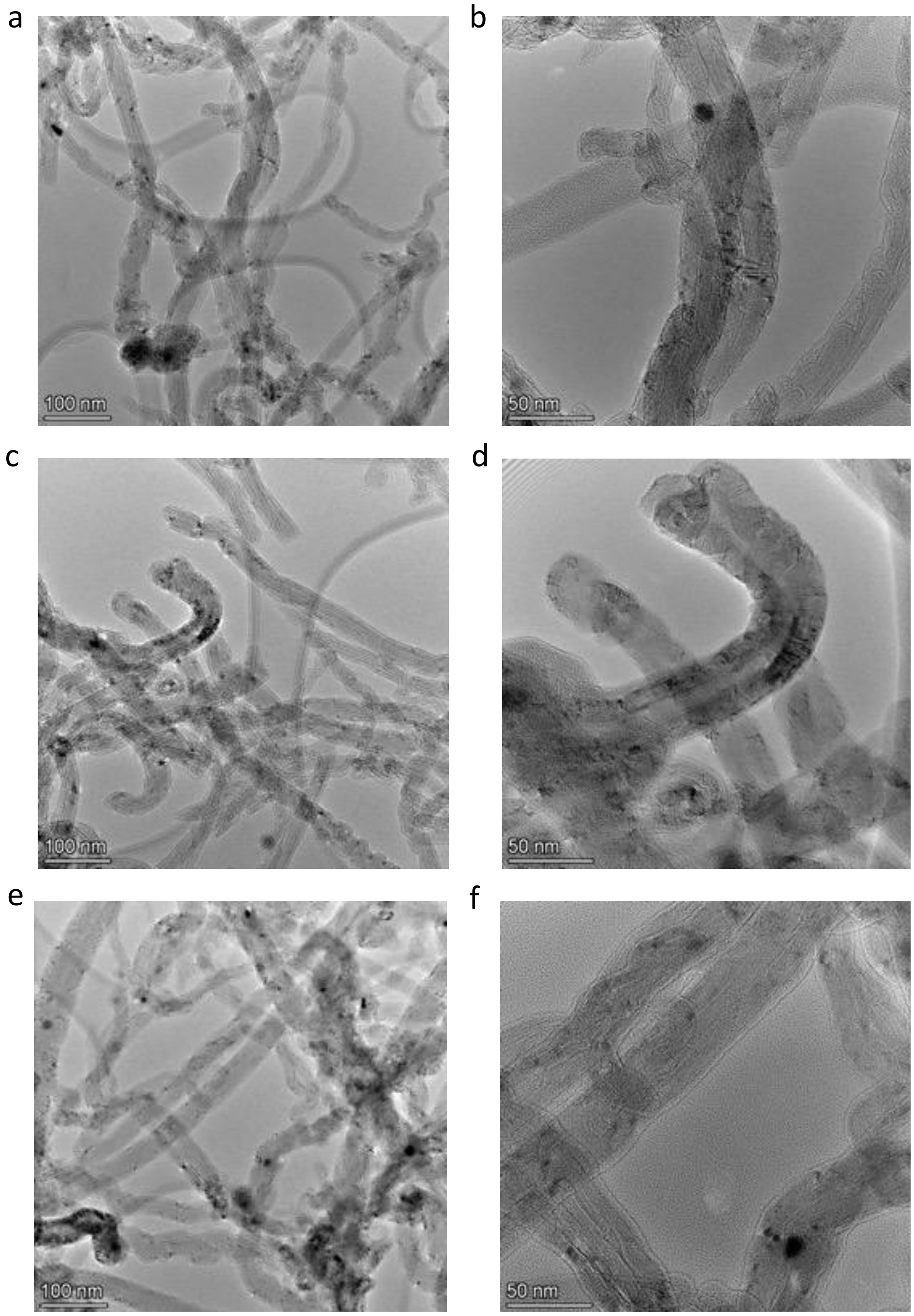

Figure S1. TEM images of (a-b) Raw-CNT, (c-d) CNT-Mel, and (e-f) CNT-Heat (Figure S1a is reproduced with permission from reference (1).[1] Copyright 2023 Elsevier).

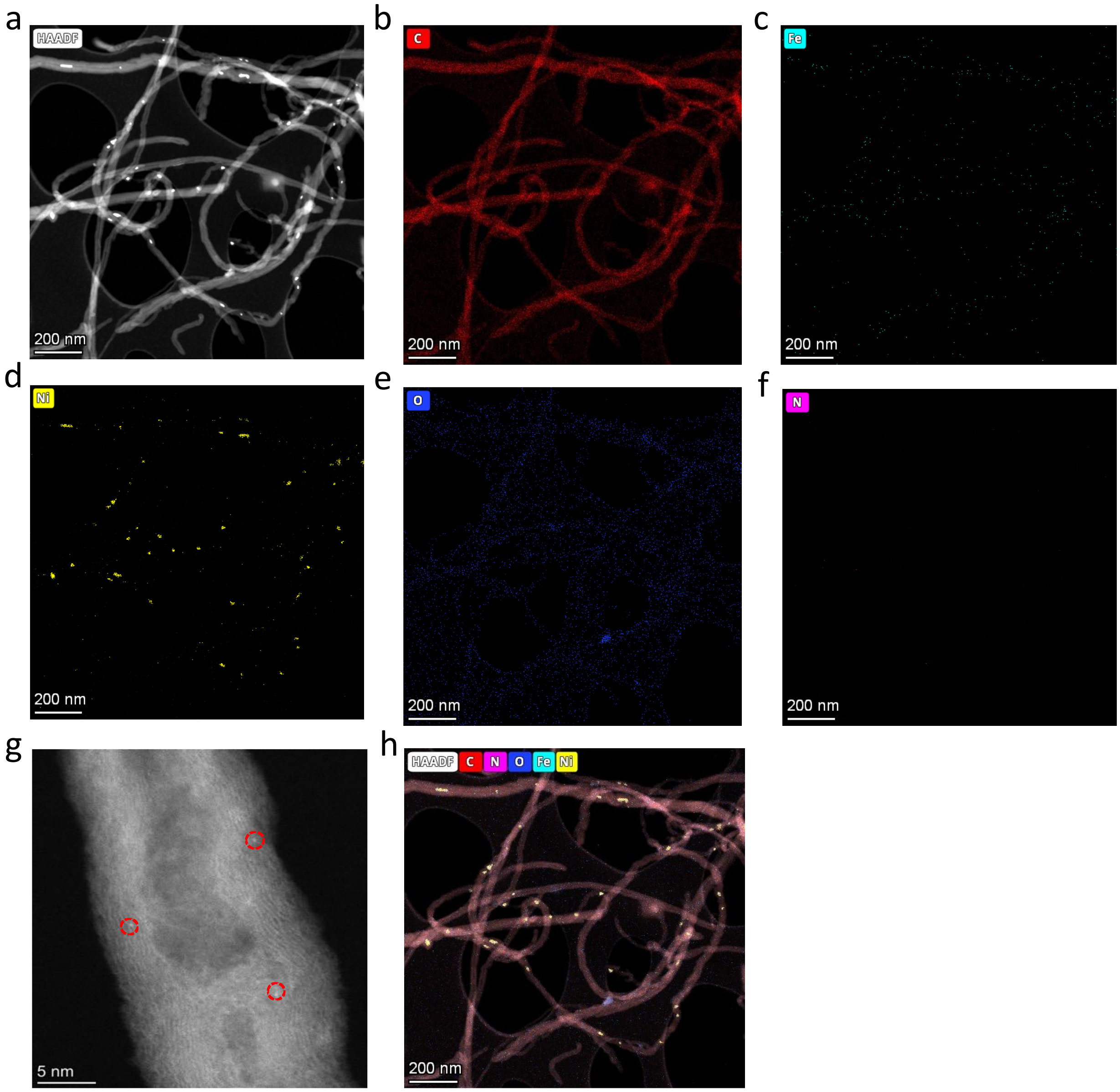

Figure S2. (a) HAADF-STEM image, elemental mapping images of (b) C, (c) Fe, (d) Ni, (e) O, (f) N of Raw-CNT, and (g-h) high-resolution STEM image of Raw-CNT.

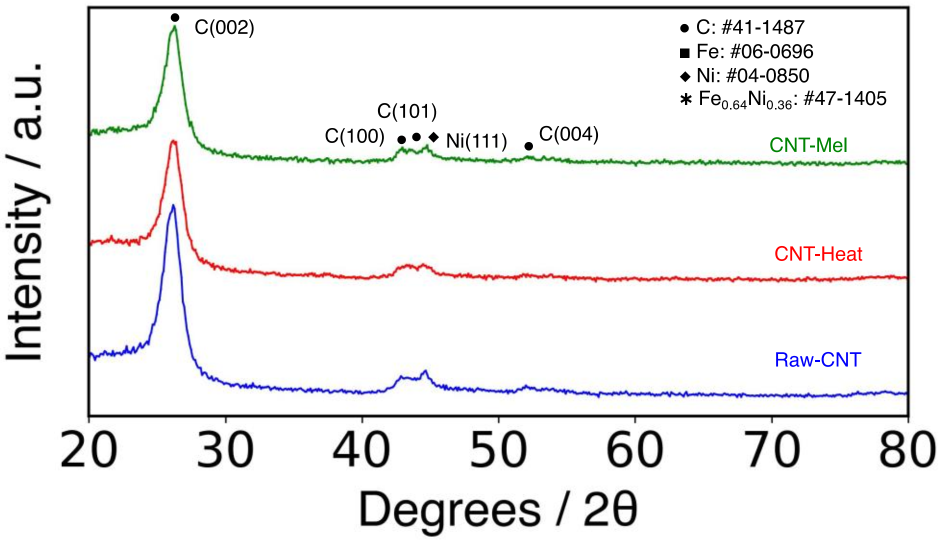

Figure S3. XRD spectra of Raw-CNT, CNT-Heat, and CNT-Mel.

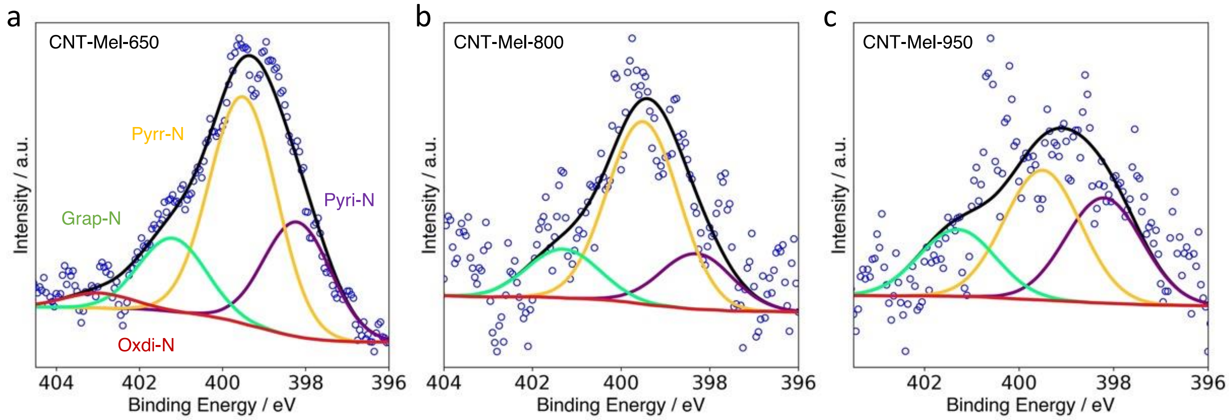

Figure S4. XPS N 1s spectra and fitting of CNT-Mel synthesized at different temperature: (a) 650 °C, (b) 800 °C, and (c) 950 °C.

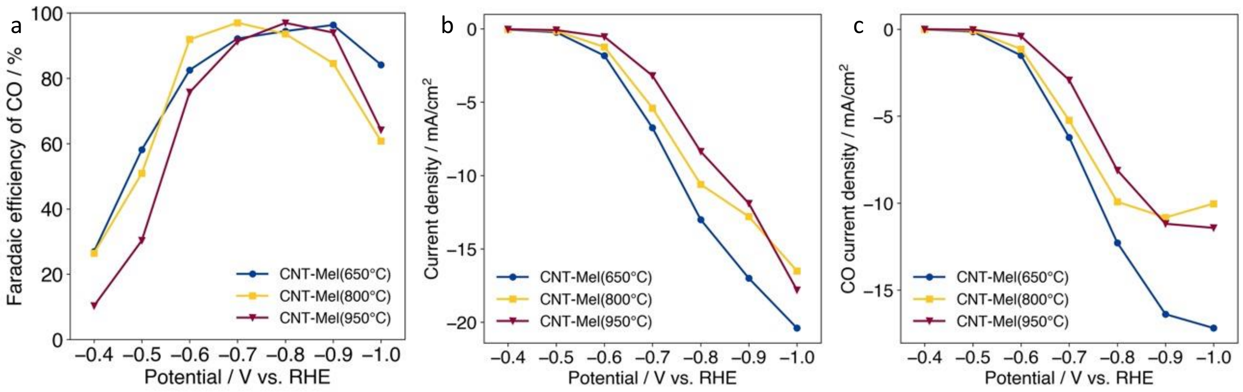

Figure S5. (a) Faradaic efficiency of CO, (b) total current density, and (c) partial CO current density of CNT-Mel synthesized at different pyrolysis temperatures in H-Cell (Electrolyte: 0.5 M $KHCO_3$).

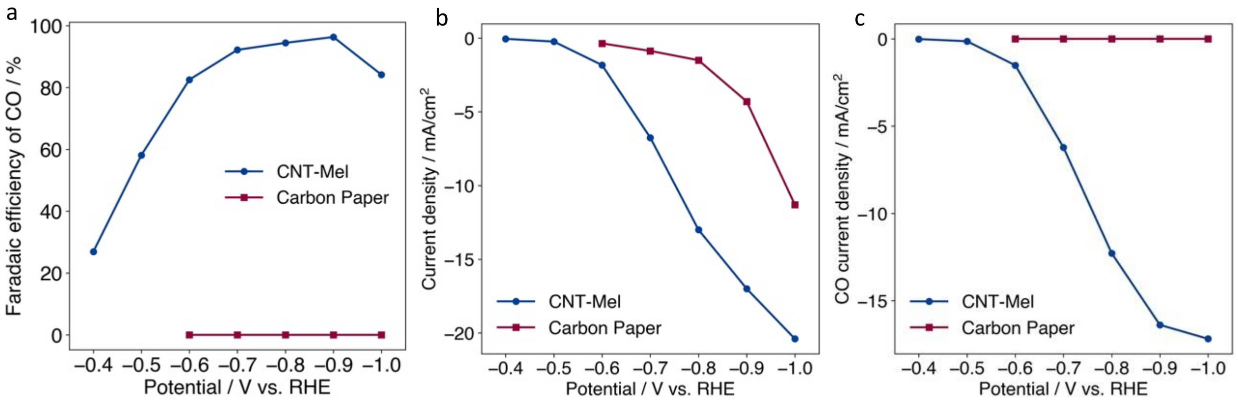

Figure S6. (a) Faradaic efficiency of CO, (b) total current density, and (C) CO current density of CNT-Mel and plain carbon paper in H-Cell (Electrolyte: 0.5 M $KHCO_3$).

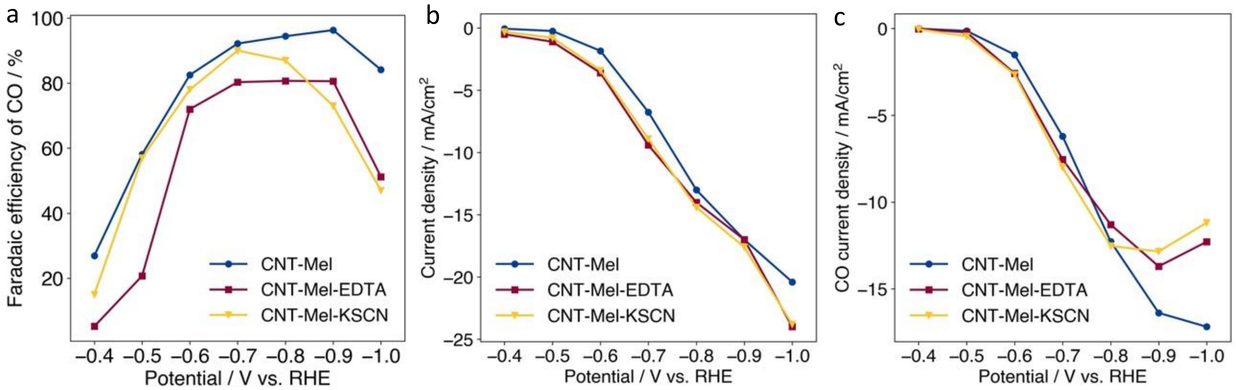

Figure S7. (a) Faradaic efficiency of CO, (b) current density, and (c) CO partial current density of CNT-Mel and samples poisoned by EDTA and KSCN (0.05 M EDTA or KSCN in 0.5 M $KHCO_3$ electrolyte) in H-Cell.

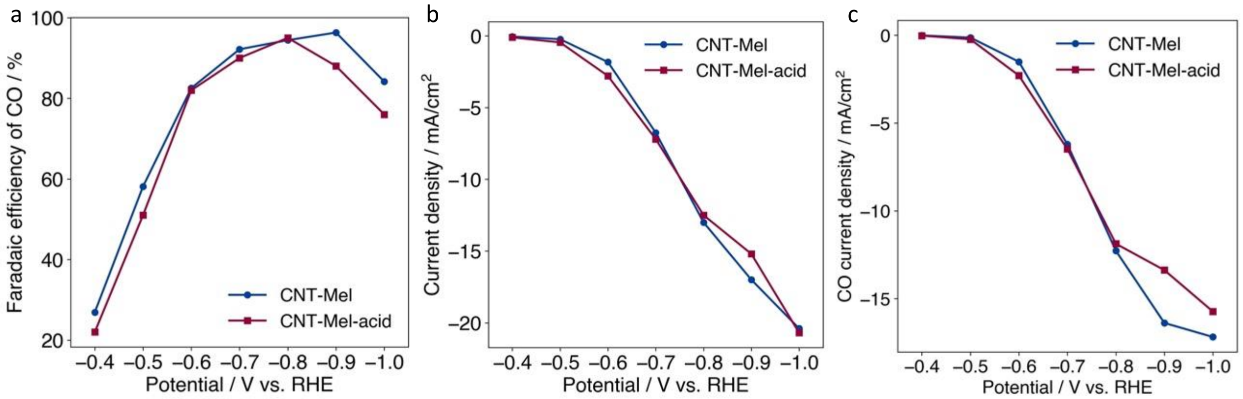

Figure S8. (a) Faradaic efficiency of CO, (b) current density, and (c) CO partial current density of CNT-Mel and CNT-Mel-acid in H-Cell (Electrolyte: 0.5 M $KHCO_3$).

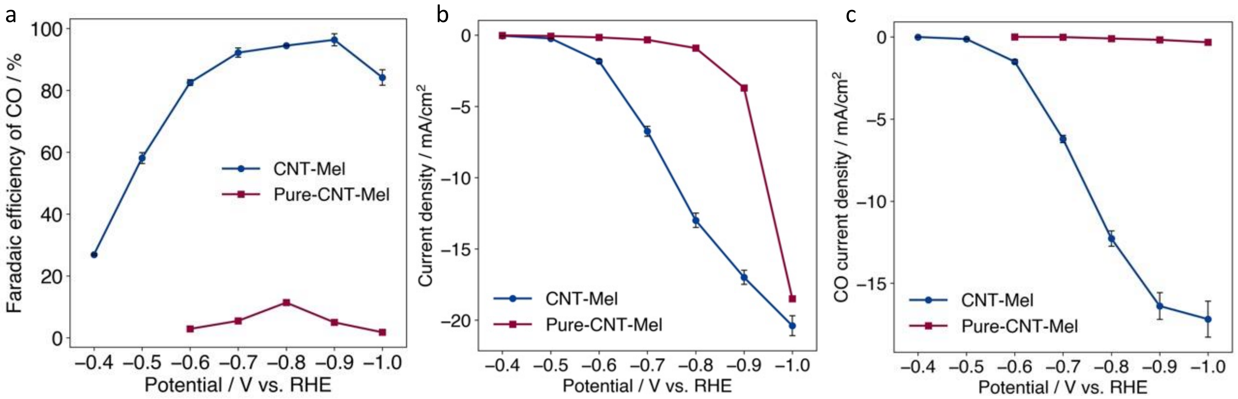

Figure S9. (a) Faradaic efficiency of CO, (b) current density, and (c) CO partial current density of CNT-Mel and Pure-CNT-Mel in H-Cell (Electrolyte: 0.5 M $KHCO_3$).

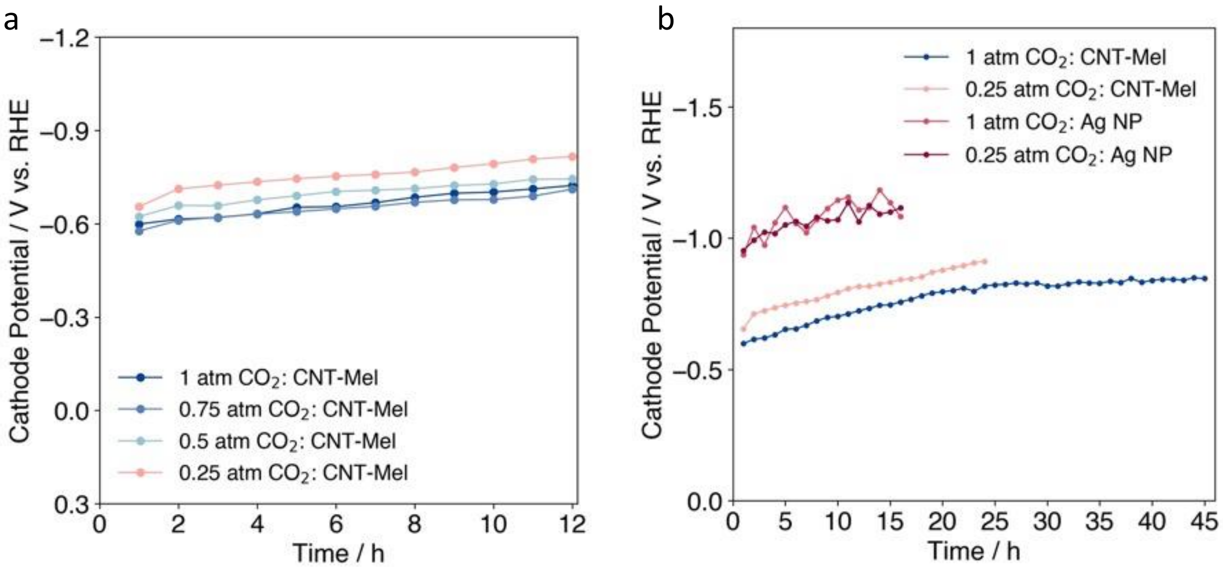

Figure S10. Cathode potential (without iR compensation) during stability test: (a) different CO_2 partial pressure and (b) comparison to Ag NP (Cell Configuration: flow cell; Electrolyte: 1 M KOH; Current density: 100 mA/cm^2).

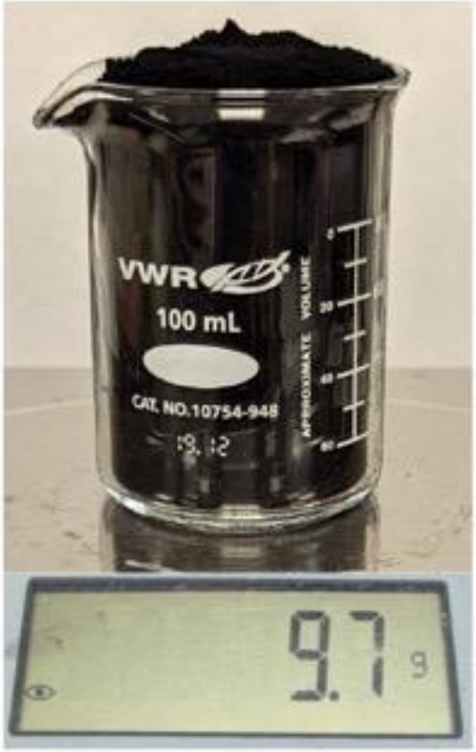

Figure S11. One batch synthesis of approximately 10 g (150 ml) of CNT-Mel.

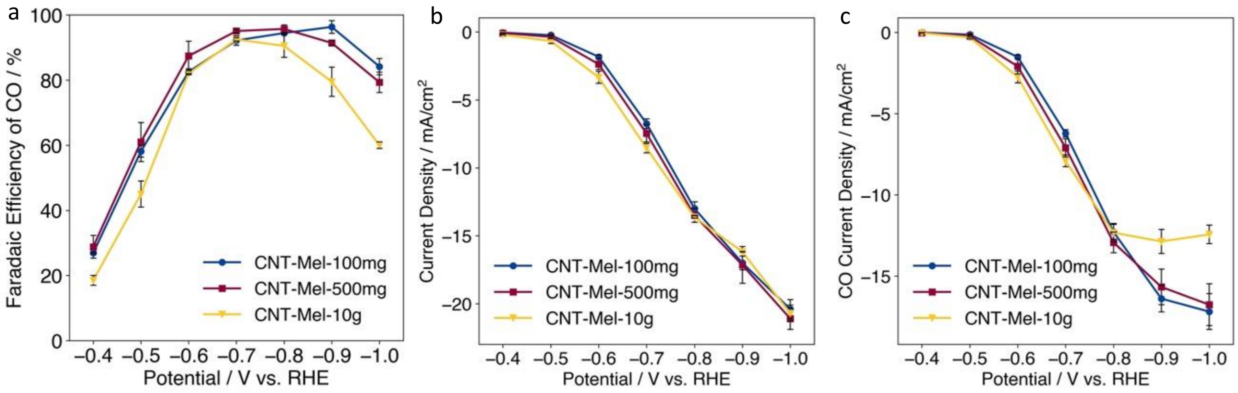

Figure S12. (a) Faradaic efficiency, (b) total current density, and (c) CO current density of CNT-Mel-100mg, CNT-Mel-500mg, and CNT-Mel-10g in the H-Cell (Electrolyte: 0.5 M $KHCO_3$).

Table S1. Surface nitrogen and metal concentration detected by XPS.

**Catalyst**	**N concentration (at. %)**	**Ni concentration (at. %)**	**Fe concentration (at. %)**
**CNT-Mel (650 °C)**	1.3	~0.1%	0.4
**CNT-Mel (800 °C)**	0.7	Not detected	0.5
**CNT-Mel (950 °C)**	0.6	Not detected	0.4
**Raw-CNT**	Not detected	~0.1%	0.5

Table S2. Comparison between the catalyst in this work with state-of-the-art Fe-based/Ni-based catalysts in the H-Cell.

Catalyst	Pre-treat	Post-wash	Metal precursor	Synthesis condition	FE(CO)	Jco (mA/cm^2)	Potential (V vs RHE)	$KHCO_3$ (M)
CNT-Mel	No	No	No	650 °C	94%	12.3	-0.8	0.5
$Fe_{0.5}d$[2]	ZIF-8 Synthesis	Yes	Yes	1050 °C	80% (50%)	4.5 (6)	-0.5 (-0.8)	0.5
$Fe-N_5$[3]	GO synthesis	Yes	Yes	800 °C	97% (50%)	2 (4.5)	-0.46 (-0.8)	0.1
Fe-N/O-C[4]	Freeze drying	Yes	Yes	1000 °C	96% (80%)	5.6 (12)	-0.57 (-0.77)	0.1
Fe-NS-C[5]	Polymerization	Yes	Yes	900 °C, twice	93%	12.1	-0.56	0.5
Fe-N-C[6]	Silica template	Yes	Yes	1000 °C, twice	81% (50%)	2.8 (4)	-0.57 (-0.8)	0.5
Fe-N-C[7]	ZIF-8	No	Yes	1000 °C	~90% (40%)	~1 (~2)	-0.5 (-0.8)	0.5
SMFeSCN[8]	SBA-15 template	Yes	Yes	900 °C, twice	99% (60%)	~3.5 (~11)	-0.55 (-0.8)	0.1
Fe-N-PC[9]	Strong acid/oxidant	No	Yes	900 °C	63% (20%)	3.2 (~5)	-0.5 (-0.8)	0.5
NC@Fe[10]	Wet impregnation	Yes	Yes	800 °C	90.6% (~45%)	0.75 (~1.1)	-0.6 (-0.8)	0.1
Fe-N-CNT@GNR-2[11]	Strong acid/oxidant	No	No	900 °C	95%	10.7	-0.76	0.1
Fe-CNPs[12]	Silica template/ZIF	Yes	Yes	1000 °C	98.8% (~80%)	~6 (~11)	-0.58 (-0.78)	1
Fe-N-G-p[13]	GO synthesis/H_2O_2 etching	No	Yes	900 °C	94% (~60%)	4.3 (6.5)	-0.58 (~-0.75)	0.1
Fe-N-C[14]	ZIF, washing	No	Yes	1000 °C	~90% (~50%)	~5 (~9)	-0.5 (-0.8)	0.5
Fe/NG[15]	GO synthesis, freeze drying, etc.	No	Yes	750 °C	80% (60%)	~1.5 (~2.5)	-0.6 (-0.8)	0.1
NC-CNT(Ni)[16]	Polymerization, hydrothermal	No	No	800 °C	90%	~7	-0.8	0.1
Ni-PACN[17]	Polymerization	No	Yes	900 °C	99%	-21	-1.1	0.1
Ni/NC[18]	Wet impregnation	No	Yes	950 °C	92.3%	-5	-0.8	0.1
Ni-NCH-1000[18]	Silica template	Yes	Yes	1000 °C	94%	-25.4	-0.9	0.1
Ni-N-C[19]	Silica template	Yes	Yes	1000 °C	87%	-1.5	-0.57	0.5
Ni1000[20]	Polymerization	Yes	Yes	1000 °C	90%	-4.4	-0.8	0.1

Ni-N/CNT-50[21]	MOF synthesis	Yes	Yes	800 °C	~100	-22	-0.8	0.5
Ni-SAs[22]	MOF synthesis	Yes	Yes	1000 °C	97	~-8	-0.8	0.5
Ni-NG[23]	GO synthesis, wet impregnation	No	Yes	750 °C	95	-11	-0.75	0.5
Ni-N-C[24]	Wet impregnation, hydrothermal	Yes	Yes	900 °C	~80	-12	-0.85	0.1

Table S3. Comparison of the mass of catalysts synthesized in one batch for high-performing Fe or Ni-based M-N-C catalysts in the literature.

Catalyst	Carbon Precursor	One-Batch Catalyst Mass	Literature
CNT-Mel-10g	CNT	~ 10 g	This work
FeNC	Carbon black	0.4 g	Chem. Sci., 2018, 9, 5064-5073[25]
Ni SAs/NCNTs	ZIF-8	0.2 g	Applied Catalysis B: Environmental 241 (2019) 113–119[26]
Fe-N/CNT@GNR-2	CNT	0.5 g	ACS Nano 2020, 14, 5, 5506–5516[27]
Fe-N-C	Carbon black	0.4 g	Energy Environ. Sci., 2019,12, 640-647[24]
Fe/NG-750	Graphene	0.2 g	Adv. Energy Mater. 2018, 1703487[28]
Fe-N-C	ZIF-8	0.4 g	J. Am. Chem. Soc. 2019, 141, 31, 12372–12381[29]
Ni@NiNCM	o-phenylendiamine	0.25 g	Angew. Chem. 2021, 133, 12066 – 12072[30]
NiSA/N-C	g-C_3N_4	0.05 g	Nano Energy 77 (2020) 105158[31]
Ni-N-C	Graphene quantum dots	0.09 g	Nature Chemistry volume 13, pages887–894 (2021)[32]
FeNC/NiNC	ZIF-8	0.4 g	ACS Catal. 2019, 9, 11, 10426–10439[33]
Fe-NC SAC	Porous carbon	0.06 g	Nature communications 10.1 (2019): 1-11[34]
Fe-N-G-p	Graphene	0.1 g	ACS Catal. 2020, 10, 19, 10803–10811[35]

Note: most of the literature did not directly report one-batch mass of catalyst, however, the maximum amount of catalyst can be calculated based on the precursor composition and pyrolysis temperature since the nitrogen precursors usually completely decompose during the high-temperature pyrolysis (> 800 °C) and metal contents in the form of single atoms do not contribute much to overall catalyst mass (usually less than 5 wt. %). Typically, the mass of carbon from the precursor determines the final mass of the catalyst. In **Table S3**, the one-batch catalyst mass is calculated based on the following conditions: (1) when the carbon precursor is

carbon allotropes (e.g., CNT, carbon black, etc.), the catalyst mass is roughly equal to the mass of carbon precursor; (2) when the carbon precursor is organic materials (e.g., ZIF-8), the actual catalyst mass would be significantly smaller than the carbon precursor mass, because organic materials decompose significantly during carbonization process, and as a result, an estimated 50% mass conversion from precursor to catalyst is applied according to the literature.[36]

Table S4. Comparison between the catalyst in this work with state-of-the-art Fe-based/Ni-based M-N-C catalysts in the flow cell when running long-term tests.

Catalyst	Electrolyte	CO selectivity (%)	Jco (mA/cm^2)	Potential (V vs. RHE)	Stability (h)
CNT-Mel (100% CO_2)	1 M KOH	99	99	-0.78	45
CNT-Mel (25% CO_2)	1 M KOH	94	94	-0.8	24
Fe-SAs (100% CO_2)[37]	0.5 M $KHCO_3$	99.5	30	-0.9	18
Fe-N-C (100% CO_2)[24]	1 M $KHCO_3$	20	20	-0.8	N/A
Ni-N-C (100% CO_2)[24]	1 M $KHCO_3$	80	160	-0.8	20
Ni-SAC (100% CO_2)[38]	0.5 M $KHCO_3$	90	20	-0.8	N/A
NiPcP (100% CO_2)[39]	1 M KOH	99.13	197	-0.5	N/A
Ni-N-C[40]	0.5 M $KHCO_3$ (C) /2 M KOH (A)	90	90	-1.0	24
Fe-N-C[40]	0.5 M $KHCO_3$ (C) /2 M KOH (A)	>95	>95	-0.83	24
Ni-N-AC-B1[41]	1 M $KHCO_3$ (C) /2 M KOH (A)	80	40	N/A	24
Fe/N-C[42]	1 M KOH	70	1.4	N/A	N/A
Ni/N-C[42]	1 M KOH	80	80	N/A	N/A
Ni-N-C_{1000}[43]	0.5 M $KHCO_3$	95	20	-1.3	7
Ni-NCB[44]	0.1 M $KHCO_3$	~99	~90	N/A	20

Reference:

1. Gang, Y.; Li, B.; Fang, S.; Pellessier, J.; Fang, L.; Pan, F.; Du, Z.; Hu, Y. H.; Li, T.; Wang, G., Efficient electrochemical CO2 reduction to CO by metal and nitrogen co-doped carbon catalysts derived from pharmaceutical wastes adsorbed on commercial carbon nanotubes. *Chemical Engineering Journal* **2023,** *453*, 139712. (https://doi.org/10.1016/j.cej.2022.139712)
2. Huan, T. N.; Ranjbar, N.; Rousse, G.; Sougrati, M.; Zitolo, A.; Mougel, V.; Jaouen, F.; Fontecave, M., Electrochemical Reduction of CO2 Catalyzed by Fe-N-C Materials: A Structure–Selectivity Study. *ACS Catalysis* **2017,** *7* (3), 1520-1525. (https://doi.org/10.1021/acscatal.6b03353)
3. Zhang, H.; Li, J.; Xi, S.; Du, Y.; Hai, X.; Wang, J.; Xu, H.; Wu, G.; Zhang, J.; Lu, J.; Wang, J., A Graphene-Supported Single-Atom FeN5 Catalytic Site for Efficient Electrochemical CO2 Reduction. *Angewandte Chemie International Edition* **2019,** *58* (42), 14871-14876. (https://doi.org/10.1002/ange.201906079)
4. Wang, X.; Pan, Y.; Ning, H.; Wang, H.; Guo, D.; Wang, W.; Yang, Z.; Zhao, Q.; Zhang, B.; Zheng, L.; Zhang, J.; Wu, M., Hierarchically micro- and meso-porous Fe-N4O-doped carbon as robust electrocatalyst for CO2 reduction. *Applied Catalysis B: Environmental* **2020,** *266*, 118630. (https://doi.org/10.1016/j.apcatb.2020.118630)
5. Pan, F.; Li, B.; Sarnello, E.; Hwang, S.; Gang, Y.; Feng, X.; Xiang, X.; Adli, N. M.; Li, T.; Su, D.; Wu, G.; Wang, G.; Li, Y., Boosting CO2 reduction on Fe-N-C with sulfur incorporation: Synergistic electronic and structural engineering. *Nano Energy* **2020,** *68*, 104384. (https://doi.org/10.1016/j.nanoen.2019.104384)
6. Hu, X.-M.; Hval, H. H.; Bjerglund, E. T.; Dalgaard, K. J.; Madsen, M. R.; Pohl, M.-M.; Welter, E.; Lamagni, P.; Buhl, K. B.; Bremholm, M.; Beller, M.; Pedersen, S. U.; Skrydstrup, T.; Daasbjerg, K., Selective CO2 Reduction to CO in Water using Earth-Abundant Metal and Nitrogen-Doped Carbon Electrocatalysts. *ACS Catal.* **2018,** *8* (7), 6255-6264. (https://doi.org/10.1021/acscatal.8b01022)
7. Ren, W.; Tan, X.; Yang, W.; Jia, C.; Xu, S.; Wang, K.; Smith, S. C.; Zhao, C., Isolated Diatomic Ni-Fe Metal–Nitrogen Sites for Synergistic Electroreduction of CO2. *Angewandte Chemie International Edition* **2019,** *58* (21), 6972-6976. (https://doi.org/10.1002/anie.201901575)
8. Yang, H.-J.; Zhang, X.; Hong, Y.-H.; Sari, H. M. K.; Zhou, Z.-Y.; Sun, S.-G.; Li, X.-F., Superior Selectivity and Tolerance towards Metal-Ion Impurities of a Fe/N/C Catalyst for CO2 Reduction. *ChemSusChem* **2019,** *12* (17), 3988-3995. (https://doi.org/10.1002/cssc.201901330)
9. Gang, Y.; Pan, F.; Fei, Y.; Du, Z.; Hu, Y. H.; Li, Y., Highly Efficient Nickel, Iron, and Nitrogen Codoped Carbon Catalysts Derived from Industrial Waste Petroleum Coke for Electrochemical CO2 Reduction. *ACS Sustainable Chem. Eng.* **2020,** *8* (23), 8840-8847. (https://doi.org/10.1021/acssuschemeng.0c03054)
10. Peng, Z.; Huang, Y.; Wang, J.; Yang, R.; Xie, J.; Wang, Y., Metal-Modulated Nitrogen-Doped Carbon Electrocatalyst for Efficient Carbon Dioxide Reduction. *ChemElectroChem* **2020,** *7* (5), 1142-1148. (https://doi.org/10.1002/celc.202000185)
11. Pan, F.; Li, B.; Sarnello, E.; Fei, Y.; Gang, Y.; Xiang, X.; Du, Z.; Zhang, P.; Wang, G.; Nguyen, H. T.; Li, T.; Hu, Y. H.; Zhou, H.-C.; Li, Y., Atomically Dispersed Iron–Nitrogen Sites on Hierarchically Mesoporous Carbon Nanotube and Graphene Nanoribbon Networks for CO2 Reduction. *ACS Nano* **2020,** *14* (5), 5506-5516. (https://doi.org/10.1021/acsnano.9b09658)

12. Hu, C.; Bai, S.; Gao, L.; Liang, S.; Yang, J.; Cheng, S.-D.; Mi, S.-B.; Qiu, J., Porosity-Induced High Selectivity for CO2 Electroreduction to CO on Fe-Doped ZIF-Derived Carbon Catalysts. *ACS Catalysis* **2019,** *9* (12), 11579-11588. (https://doi.org/10.1021/acscatal.9b03175)
13. Pan, F.; Li, B.; Sarnello, E.; Fei, Y.; Feng, X.; Gang, Y.; Xiang, X.; Fang, L.; Li, T.; Hu, Y. H.; Wang, G.; Li, Y., Pore-Edge Tailoring of Single-Atom Iron–Nitrogen Sites on Graphene for Enhanced CO2 Reduction. *ACS Catalysis* **2020,** *10* (19), 10803-10811. (https://doi.org/10.1021/acscatal.0c02499)
14. Qin, X.; Zhu, S.; Xiao, F.; Zhang, L.; Shao, M., Active Sites on Heterogeneous Single-Iron-Atom Electrocatalysts in CO2 Reduction Reaction. *ACS Energy Lett.* **2019,** *4* (7), 1778-1783. (https://doi.org/10.1021/acsenergylett.9b01015)
15. Zhang, C.; Yang, S.; Wu, J.; Liu, M.; Yazdi, S.; Ren, M.; Sha, J.; Zhong, J.; Nie, K.; Jalilov, A. S.; Li, Z.; Li, H.; Yakobson, B. I.; Wu, Q.; Ringe, E.; Xu, H.; Ajayan, P. M.; Tour, J. M., Electrochemical CO2 Reduction with Atomic Iron-Dispersed on Nitrogen-Doped Graphene. *Advanced Energy Materials* **2018,** *8* (19), 1703487. (https://doi.org/10.1002/aenm.201703487)
16. Fan, Q.; Hou, P.; Choi, C.; Wu, T. S.; Hong, S.; Li, F.; Soo, Y. L.; Kang, P.; Jung, Y.; Sun, Z., Activation of Ni particles into single Ni–N atoms for efficient electrochemical reduction of CO2. *Advanced Energy Materials* **2020,** *10* (5), 1903068. (https://doi.org/10.1002/aenm.201903068)
17. Koshy, D. M.; Chen, S.; Lee, D. U.; Stevens, M. B.; Abdellah, A. M.; Dull, S. M.; Chen, G.; Nordlund, D.; Gallo, A.; Hahn, C., Understanding the origin of highly selective CO2 electroreduction to CO on Ni, N‐doped carbon catalysts. *Angewandte Chemie International Edition* **2020,** *59* (10), 4043-4050. (https://doi.org/10.1002/anie.201912857)
18. Li, F.; Hong, S.; Wu, T.-S.; Li, X.; Masa, J.; Soo, Y.-L.; Sun, Z., Atomically Dispersed Nickel Sites for Selective Electroreduction of CO2. *ACS Applied Energy Materials* **2019,** *2* (12), 8836-8842. (https://doi.org/10.1021/acsaem.9b01828)
19. Hu, X.-M.; Hval, H. H.; Bjerglund, E. T.; Dalgaard, K. J.; Madsen, M. R.; Pohl, M.-M.; Welter, E.; Lamagni, P.; Buhl, K. B.; Bremholm, M., Selective CO2 reduction to CO in water using earth-abundant metal and nitrogen-doped carbon electrocatalysts. *ACS catalysis* **2018,** *8* (7), 6255-6264. (https://doi.org/10.1021/acscatal.8b01022)
20. Wang, Z. L.; Choi, J.; Xu, M.; Hao, X.; Zhang, H.; Jiang, Z.; Zuo, M.; Kim, J.; Zhou, W.; Meng, X., Optimizing Electron Densities of Ni‐N‐C Complexes by Hybrid Coordination for Efficient Electrocatalytic CO2 Reduction. *ChemSusChem* **2020,** *13* (5), 929-937. (https://doi.org/10.1002/cssc.201903427)
21. Hou, Y.; Liang, Y.-L.; Shi, P.-C.; Huang, Y.-B.; Cao, R., Atomically dispersed Ni species on N-doped carbon nanotubes for electroreduction of CO2 with nearly 100% CO selectivity. *Applied Catalysis B: Environmental* **2020,** *271*, 118929. (https://doi.org/10.1016/j.apcatb.2020.118929)
22. Li, Z.; He, D.; Yan, X.; Dai, S.; Younan, S.; Ke, Z.; Pan, X.; Xiao, X.; Wu, H.; Gu, J., Size‐dependent nickel‐based electrocatalysts for selective CO2 reduction. *Angewandte Chemie* **2020,** *132* (42), 18731-18736. (https://doi.org/10.1002/ange.202000318)
23. Jiang, K.; Siahrostami, S.; Zheng, T.; Hu, Y.; Hwang, S.; Stavitski, E.; Peng, Y.; Dynes, J.; Gangisetty, M.; Su, D., Isolated Ni single atoms in graphene nanosheets for high-performance CO 2 reduction. *Energ Environ Sci* **2018,** *11* (4), 893-903. (https://doi.org/10.1039/C7EE03245E)

24. Möller, T.; Ju, W.; Bagger, A.; Wang, X.; Luo, F.; Thanh, T. N.; Varela, A. S.; Rossmeisl, J.; Strasser, P., Efficient CO 2 to CO electrolysis on solid Ni–N–C catalysts at industrial current densities. *Energ Environ Sci* **2019,** *12* (2), 640-647. (https://doi.org/10.1039/C8EE02662A)
25. Leonard, N.; Ju, W.; Sinev, I.; Steinberg, J.; Luo, F.; Varela, A. S.; Cuenya, B. R.; Strasser, P., The chemical identity, state and structure of catalytically active centers during the electrochemical CO 2 reduction on porous Fe–nitrogen–carbon (Fe–N–C) materials. *Chemical science* **2018,** *9* (22), 5064-5073. (https://doi.org/10.1039/C8SC00491A)
26. Lu, P.; Yang, Y.; Yao, J.; Wang, M.; Dipazir, S.; Yuan, M.; Zhang, J.; Wang, X.; Xie, Z.; Zhang, G., Facile synthesis of single-nickel-atomic dispersed N-doped carbon framework for efficient electrochemical CO2 reduction. *Applied Catalysis B: Environmental* **2019,** *241*, 113-119. (https://doi.org/10.1016/j.apcatb.2018.09.025)
27. Pan, F.; Li, B.; Sarnello, E.; Fei, Y.; Gang, Y.; Xiang, X.; Du, Z.; Zhang, P.; Wang, G.; Nguyen, H. T., Atomically dispersed iron–nitrogen sites on hierarchically mesoporous carbon nanotube and graphene nanoribbon networks for CO2 reduction. *ACS Nano* **2020,** *14* (5), 5506-5516. (https://doi.org/10.1021/acsnano.9b09658)
28. Zhang, C.; Yang, S.; Wu, J.; Liu, M.; Yazdi, S.; Ren, M.; Sha, J.; Zhong, J.; Nie, K.; Jalilov, A. S., Electrochemical CO2 reduction with atomic iron‐dispersed on nitrogen‐doped graphene. *Advanced Energy Materials* **2018,** *8* (19), 1703487. (https://doi.org/10.1002/aenm.201703487)
29. Sun, Y.; Silvioli, L.; Sahraie, N. R.; Ju, W.; Li, J.; Zitolo, A.; Li, S.; Bagger, A.; Arnarson, L.; Wang, X., Activity–selectivity trends in the electrochemical production of hydrogen peroxide over single-site metal–nitrogen–carbon catalysts. *Journal of the American Chemical Society* **2019,** *141* (31), 12372-12381. (https://doi.org/10.1021/jacs.9b05576)
30. Wang, X.; Sang, X.; Dong, C. L.; Yao, S.; Shuai, L.; Lu, J.; Yang, B.; Li, Z.; Lei, L.; Qiu, M., Proton capture strategy for enhancing electrochemical CO2 reduction on atomically dispersed metal–nitrogen active sites. *Angewandte Chemie* **2021,** *133* (21), 12066-12072. (https://doi.org/10.1002/ange.202100011)
31. Lu, Y.; Wang, H.; Yu, P.; Yuan, Y.; Shahbazian-Yassar, R.; Sheng, Y.; Wu, S.; Tu, W.; Liu, G.; Kraft, M., Isolated Ni single atoms in nitrogen doped ultrathin porous carbon templated from porous g-C3N4 for high-performance CO2 reduction. *Nano Energy* **2020,** *77*, 105158. (https://doi.org/10.1016/j.nanoen.2020.105158)
32. Xia, C.; Qiu, Y.; Xia, Y.; Zhu, P.; King, G.; Zhang, X.; Wu, Z.; Kim, J. Y. T.; Cullen, D. A.; Zheng, D., General synthesis of single-atom catalysts with high metal loading using graphene quantum dots. *Nature chemistry* **2021,** *13* (9), 887-894. (https://doi.org/10.1038/s41557-021-00734-x)
33. Li, J.; Pršlja, P.; Shinagawa, T.; Martin Fernandez, A. J.; Krumeich, F.; Artyushkova, K.; Atanassov, P.; Zitolo, A.; Zhou, Y.; García-Muelas, R., Volcano trend in electrocatalytic CO2 reduction activity over atomically dispersed metal sites on nitrogen-doped carbon. *ACS Catalysis* **2019,** *9* (11), 10426-10439. (https://doi.org/10.1021/acscatal.9b02594)
34. Zhao, L.; Zhang, Y.; Huang, L.-B.; Liu, X.-Z.; Zhang, Q.-H.; He, C.; Wu, Z.-Y.; Zhang, L.-J.; Wu, J.; Yang, W., Cascade anchoring strategy for general mass production of high-loading single-atomic metal-nitrogen catalysts. *Nature communications* **2019,** *10* (1), 1-11. (https://doi.org/10.1038/s41467-019-09290-y)

35. Pan, F.; Li, B.; Sarnello, E.; Fei, Y.; Feng, X.; Gang, Y.; Xiang, X.; Fang, L.; Li, T.; Hu, Y. H., Pore-edge tailoring of single-atom iron–nitrogen sites on graphene for enhanced CO2 reduction. *ACS Catal.* **2020,** *10* (19), 10803-10811. (https://doi.org/10.1021/acscatal.0c02499)
36. Gadipelli, S.; Guo, Z. X., Tuning of ZIF‐derived carbon with high activity, nitrogen functionality, and yield–a case for superior CO2 capture. *ChemSusChem* **2015,** *8* (12), 2123-2132. (https://doi.org/10.1002/cssc.201403402)
37. Xie, M.; Gao, N.; Xiao, G.; Ge, M.; Du, X.-L.; Mei, B.; Wang, J.-Q.; Li, T., Size-dependent selectivity of iron-based electrocatalysts for electrochemical CO 2 reduction. *Sustainable Energy & Fuels* **2022,** *6* (3), 736-743. (https://doi.org/10.1039/D1SE01726H)
38. Liu, L.; Liu, S.; Li, L.; Qi, H.; Yang, H.; Huang, Y.; Wei, Z.; Li, L.; Xu, J.; Liu, B., A general method to construct single-atom catalysts supported on N-doped graphene for energy applications. *Journal of Materials Chemistry A* **2020,** *8* (13), 6190-6195. (https://doi.org/10.1039/C9TA11715F)
39. Wei, S.; Zou, H.; Rong, W.; Zhang, F.; Ji, Y.; Duan, L., Conjugated nickel phthalocyanine polymer selectively catalyzes CO2-to-CO conversion in a wide operating potential window. *Applied Catalysis B: Environmental* **2021,** *284*, 119739. (https://doi.org/10.1016/j.apcatb.2020.119739)
40. Duarte, M.; Daems, N.; Hereijgers, J.; Arenas-Esteban, D.; Bals, S.; Breugelmans, T., Enhanced CO2 electroreduction with metal-nitrogen-doped carbons in a continuous flow reactor. *Journal of CO2 Utilization* **2021,** *50*, 101583. (https://doi.org/10.1016/j.jcou.2021.101583)
41. Daems, N.; De Mot, B.; Choukroun, D.; Van Daele, K.; Li, C.; Hubin, A.; Bals, S.; Hereijgers, J.; Breugelmans, T., Nickel-containing N-doped carbon as effective electrocatalysts for the reduction of CO 2 to CO in a continuous-flow electrolyzer. *Sustainable energy & fuels* **2020,** *4* (3), 1296-1311. (https://doi.org/10.1039/C9SE00814D)
42. Li, J.; Zitolo, A.; Garces-Pineda, F. A.; Asset, T.; Kodali, M.; Tang, P.; Arbiol, J.; Galán-Mascarós, J. R.; Atanassov, P.; Zenyuk, I. V., Metal oxide clusters on nitrogen-doped carbon are highly selective for CO2 electroreduction to CO. *ACS Catal.* **2021,** *11* (15), 10028-10042. (https://doi.org/10.1021/acscatal.1c01702)
43. Wang, W.; Chen, K.; Sun, Y.; Zhou, S.; Zhang, M.; Yuan, J., Mesoporous Ni-NC as an efficient electrocatalyst for reduction of CO2 into CO in a flow cell. *Applied Materials Today* **2022,** *29*, 101619. (https://doi.org/10.1016/j.apmt.2022.101619)
44. Zheng, T.; Jiang, K.; Ta, N.; Hu, Y.; Zeng, J.; Liu, J.; Wang, H., Large-Scale and Highly Selective CO2 Electrocatalytic Reduction on Nickel Single-Atom Catalyst. *Joule* **2019,** *3* (1), 265-278. (https://doi.org/10.1016/j.joule.2018.10.015)
